# Basal ganglia functional connectivity network analysis does not support the ‘noisy signal’ hypothesis of Parkinson’s disease

**DOI:** 10.1093/braincomms/fcad123

**Published:** 2023-04-13

**Authors:** David Williams

**Affiliations:** Department of Internal Medicine, College of Medicine and Health Sciences, United Arab Emirates University (UAEU), PO Box 15551, Al Ain, United Arab Emirates

**Keywords:** Parkinson’s disease, basal ganglia, functional connectivity, entropy, brain networks

## Abstract

The ‘noisy signal’ hypothesis of basal ganglia dysfunction in Parkinson’s disease (PD) suggests that major motor symptoms of the disorder are caused by the development of abnormal basal ganglia activity patterns resulting in the propagation of ‘noisy’ signals to target systems. While such abnormal activity patterns might be useful biomarkers for the development of therapeutic interventions, correlation between specific changes in activity and PD symptoms has been inconsistently demonstrated, and raises questions concerning the accuracy of the hypothesis. Here, we tested this hypothesis by considering three nodes of the basal ganglia network, the subthalamus, globus pallidus interna, and cortex during self-paced and cued movements in patients with PD. Interactions between these regions were analyzed using measures that assess both linear and non-linear relationships. Marked changes in the network are observed with dopamine state. Specifically, we detected functional disconnection of the basal ganglia from the cortex and higher network variability in untreated PD, but various patterns of directed functional connectivity with lower network variability in treated PD. When we examine the system output, significant correlation is observed between variability in the cortico-basal ganglia network and muscle activity variability but only in the treated state. Rather than supporting a role of the basal ganglia in the transmission of noisy signals in patients with PD, these findings suggest that cortico-basal ganglia network interactions by fault or design, in the treated Parkinsonian state, are actually associated with improved cortical network output variability.

## Teaser

Williams tests the noisy signal hypothesis of Parkinson’s disease by examining functional connectivity in the cortico-basal ganglia network and its relationship to output EMG activity in Parkinson’s disease patients. The findings do not support the hypothesis but suggest cortico-basal ganglia interaction is associated with improved cortical network output variability.

## Graphical Abstract

**Graphical abstract:**
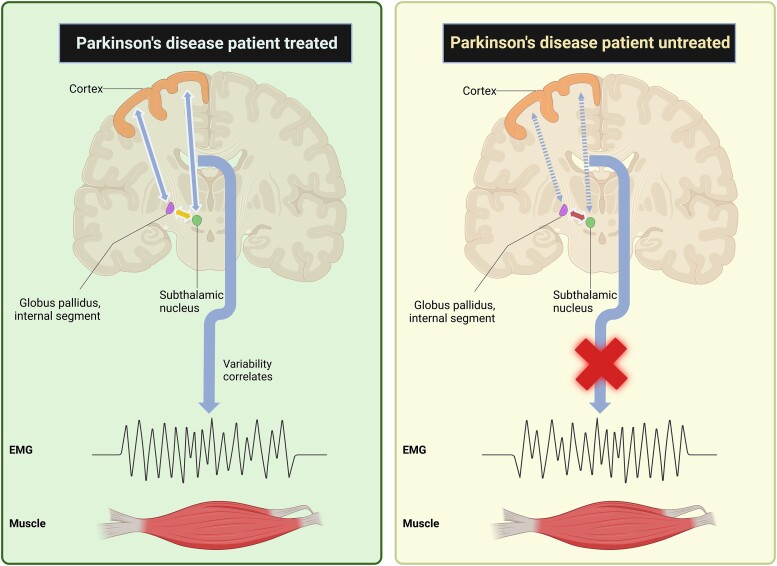

## Introduction

The basal ganglia comprise a group of interconnected subcortical nuclei including the striatum, globus pallidus interna (GPI) and externa (GPE), subthalamus (STN) and substantia nigra pars compacta and reticulata (SNr), that themselves are connected to other cortical, midbrain and brainstem regions. These nuclei display a remarkable degree of phylogenetic conservation with evidence apparent of common anatomical interconnections, and neuropharmacological signal transmission not merely between mammalian and amphibian species,^1^ but in non-mammalian vertebrates that diverged from the mammalian vertebrate evolutionary lineage hundreds of millions of years ago.^2,3^ They have also long been associated with the pathology of Parkinson’s disease (PD), and other so called hypo- and hyperkinetic movement disorders, i.e. dystonia, chorea, and hemiballism; evident through clinical lesion studies,^4,5^ and post mortem macro- and microscopic pathological changes, such as the prominent dopaminergic nigrostriatal degeneration of PD itself.^6^

Their clinical significance, coupled with the accumulation of fundamental observations concerning the neuroanatomy, neuropharmacology and neurophysiology of the nuclei, encouraged and facilitated the formulation of a seminal circuit model of basal ganglia function^7,8^ over 30 years ago, that not only explained some elements of the involvement of the basal ganglia in the pathology of hypokinetic and hyperkinetic movement disorders, but also made important predictions regarding potential beneficial therapeutic interventions in the circuit model. Fundamental components of this model were that the basal ganglia were perceived as engaged in action selection of motor and non-motor behaviours with inputs and outputs from the network connected via either a direct or indirect pathway. The direct pathway comprised connections from striatum to output GPI and SNr nuclei, with the interposition of the GPE and STN in the indirect pathway. In addition, dopamine was proposed to act differentially between the two pathways with essentially opposing effects. Disorders of the basal ganglia with hypokinetic or hyperkinetic symptoms were therefore proposed to arise from imbalance in the activation of the two pathways via effects on the level of activity of output nuclei neuronal inhibitory projections to target regions. Much supporting evidence for elements of the model has been published,^9^ and as noted above predictions derived from the model supported and encouraged the further development of neurosurgical interventions including pallidotomy, subthalamotomy and latterly deep brain stimulation (DBS) in the thalamus, GPI and STN, which have demonstrated clinical benefits in PD patients. There have, however, been long established paradoxes of the model. These include the observation that lesions of the globus pallidus may actually benefit hyperkinetic disorders such as Huntington’s Chorea,^10^ and that DBS of the GPI may relieve PD and Huntington’s disease associated dyskinesia^11,12^; both opposite to outcomes predicted. Similarly, it is observed that lesions of the motor thalamus do not exacerbate the symptoms of PD.^13^ Early attempts to explain these paradoxes posited the ‘noisy signal’ hypothesis, suggesting that abnormal patterns of basal ganglia activity rather than absolute levels or rates of neuronal activity disrupt signal transmission, and that DBS and lesion interventions alleviate or override these abnormalities.^13,14^ This has been supported by studies demonstrating abnormal patterns of neuronal activity, oscillatory burst firing,^15,16^ and increased beta band (13–30 Hz) oscillatory field potential activity,^17,18^ reduced with dopamine administration in Parkinsonian patients and animal models of Parkinsonism. There remain fundamental unresolved elements, however, such as how abnormal patterns of activity or oscillatory synchrony may both induce hypokinetic (bradykinesia) and hyperkinetic (dyskinesia) symptoms in PD; and how information transmission may actually be affected through the course of movement by such changes. There are also apparent inconsistencies such as the lack of clear correlation between features such as oscillatory synchrony and the severity of some symptoms in PD patients^19^; the lack of necessity for oscillatory local field potential (LFP) activity to be present in the symptomatic 1-methyl-4-phenyl-1,2,3,6-tetrahydropyridine-lesioned primate model,^20^ and the inability of delivered stimulation to reproduce motor deficits in a mammalian model.^21^

The current work addresses these outstanding issues, by considering three elements as paramount in basal ganglia function. Firstly, that the evolutionary conservation of basal ganglia architecture likely reflects the fact that the network properties of the structures are integral to their function rather than isolated local nuclear neuronal activity, and as such consideration of the network relationships as a whole may give insight into that function. Secondly, that information transmission in order to effect behavioural outcomes is fundamental to basal ganglia function, and as such interaction or functional connectivity between regions is necessary to effect those outcomes; and, lastly, that even in the presence of functionally interconnected basal ganglia regions any information transmission must produce an output that has a sufficiently low error rate to allow correct transmission of any signal. In an effort to clarify these elements in the context of basal ganglia function in Parkinsonian patients a data set of LFP activity including EEG and GPI, EEG and STN, and GPI and STN simultaneous recordings in a series of PD patients subsequent to deep brain stimulation electrode implantation during the performance of either spontaneous, or externally cued motor activity and in on (with anti-Parkinsonian dopaminergic treatment), and off (off anti-Parkinsonian medication) states was compiled and analyzed. Across the patient population, this allowed for consideration of 3 ‘nodes’ in the basal ganglia network, with analysis of subthalamic-globus pallidus, cortico-subthalamic, and cortico-GPI interactions; and for consideration of the effect of the dopamine medicated state on these network relationships. Functional connectivity was then considered between components of the network using methods that sought to avoid assumptions of linearity typically made in prior analyses; and quality of potential information transmission was further considered by examining the associated variability (or entropy) of these network interactions. Finally, outputs from the network were studied by analysis of the relationships of the network activity to simultaneous EMG.

## Materials and methods

### Patients and surgery

LFP data for analysis were derived from recordings made post-operatively from macroelectrodes inserted into either or both the GPI and STN of seven patients with PD (Table 1). Analysis undertaken between regions only involved data from subjects with the respective recorded data as noted in the Table, e.g. self-paced GPI–STN activity used data from subjects 3 and 5. All electrodes implanted were Medtronic 3389 with 0.5 mm spacing between contacts. All data analyzed were considered in the contralateral nuclei to the motor task undertaken (Table 1), even when bilateral STN were available. A single EEG bipole anterior to the vertex in the midline, at CzFz by the international 10–20 system was available for consideration of network relationships in four of the patients in the treated and untreated state. Patients participated with informed consent and their clinical details are otherwise noted in Table 1. All the patients were recorded in both the treated or ‘on’ state having taken their dopaminergic medication which relieves the symptoms of PD, and also recorded in the ‘off’ state without treatment. The untreated or off state was defined as being withdrawn from antiparkinsonian medication overnight; the treated state, after a period of 45–135 minutes post levodopa administration, i.e. during the clinical effective period of the drug.

**Table 1:** Clinical details of subjects

Subject no:	Age (years and sex)	Disease duration (yrs)	Location of macroelectrodes	Motor UPDRS ON/OFF	Medication (oral daily dose)	Clinical features during recording	EEG	EMG	Motor task
1	39 M	7	Right STN & GPI	15/80	Levodopa 1300 mg Ropinirole 4 mg	No rest tremor. Mild biphasic dyskinesia without dyskinesia during recording	−	−	Cued
2	68 M	16	Right STN & GPI	58/97	Apomorphine subcutaneously 5 mg/h	Tremor off Dyskinesia on	−	−	Cued
3	37 F	10	Right STN & GPI	7/65	Levodopa 150 mg Ropinirole 4 mg	No rest tremor Biphasic dyskinesia without dyskinesia during recording	+	−	Cued Self-paced
4	69 M	10	Right STN Left STN	24/35	Levodopa 800 mg Pergolide 10 mg Entacapone 1000 mg Amantadine 200 mg	N/A	+	+	Self-paced
5	64 M	9	Left STN & GPI	20/56	Levodopa 1000mg Ropinirole 6 mg	Rest tremor and akinetic rigidity off	+	+	Self-paced
6	62 M	13	Right STN Left STN	44/63	Levodopa 450 mg Cabergoline 4 mg Entacapone 200 mg	N/A	+	−	Self-paced
7	64 M	11	Right STN Left STN	18/47	Levodopa 700 mg	N/A	+	+	Self-paced

### Experimental protocol

The experimental protocol consisted of each subject moving a handheld joystick, as quickly as possible forward with return to the neutral position. Self-paced movements were undertaken at a rate determined by the subject, while externally cued were in response to a go cue, after an initial warning preparatory cue was presented 2.5 sec prior. The warning cue was a green light-emitting diode (LED) and go cue a red LED. Trials were interspersed by a quasi-randomized wait period of 17–30 sec. LFPs were recorded from macroelectrodes in the contralateral GPI and STN simultaneously, in addition to joystick position, cue/go information and EEG (in patients as noted above). EEG was recorded via silver/silver chloride electrodes. Deep brain electrode activity was recorded from adjacent pairs of bipolar macroelectrode contacts. LFPs, EMG, and EEG were filtered at 0.5–300 Hz and amplified (×100 000–500 000). Sampling was performed at 512 Hz in all subjects but one for the cued task. The latter data was downsampled to 512 Hz for further analysis. Recordings were made at 1950, 1600, 950, 1650, and 1600 Hz, respectively, for self-paced data in subjects three to seven, and down-sampled to 950 Hz for analysis. Mean reaction times for cued responses in the treated group trials was 411 ± 377 ms (2 sd); and 444 ± 314 ms in the untreated state. Mean duration of movements in the cued paradigm was 652 ± 893 ms (2 sd) in the treated state; and 915 ± 476 ms (2 sd) in the untreated state. Mean duration of movements in the self-paced movement treated state was 218 ± 235 ms (2 sd), and 654 ± 896 ms (2 sd) in the untreated group.

### Method of analysis

Each macroelectrode comprises a tetrode with contacts numbered 0 to 3, and only bipolar (i.e. 01, 12, and 23) recordings, not common averaged constructs of the LFP were analyzed to limit potential contamination from volume conducted sources. LFP and EMG data were initially visually inspected and any sections with obvious artifact (such as large amplitude deviations manifesting across all three bipoles suggesting a distant source) were removed. The optimal bipole of the given macroelectrode was selected for further analysis, based upon features suggesting the greatest likelihood of being located within the respective nucleus such as evidence of phase reversal between neighbouring bipoles. Variations in potential difference recorded by macroelectrodes associated with variation in proximity to source activity, electrode impedance or recording amplification were mitigated by *z*-score transformation standardization of the data. EMG data was similarly standardized.

Individual samples were as such represented in terms of numbers of standard deviations of the activity from the mean. Data for further analysis was limited to that encompassing four standard deviations + or - the mean, effectively representing all significant LFP activity at a given site. Data beyond four standard deviations underwent no further analysis. The activity in both the GPI and STN contralateral to the hand directed to respond by the cue signal were segmented into individual trial sections beginning 1.5 sec prior to onset of joystick movement and finishing 1.5 sec after onset of joystick movement in the self-paced protocol; and 1.5 sec prior to warning cue presentation and finishing 1.5 sec after go cue presentation, with go cue presentation occurring 2.5 sec after warning cue presentation in the externally cued protocol. In the externally cued paradigm only trials with a motor response within 700 msec were analyzed in the externally cued task. Patients 1, 2, and 3 therefore had 52, 25, and 39 trials, respectively, in the treated state, and patients 1 and 2, 23, and 34 in the untreated state. In the self-paced paradigm, patients 3, 4, 5, 6, and 7 undertook 27, 20, 13, 12, and 18 movements, respectively, in the treated state and 25, 16, 10, 10, and 15 movements in the untreated state.

### Inter-regional model derivation

**Figure 1:**
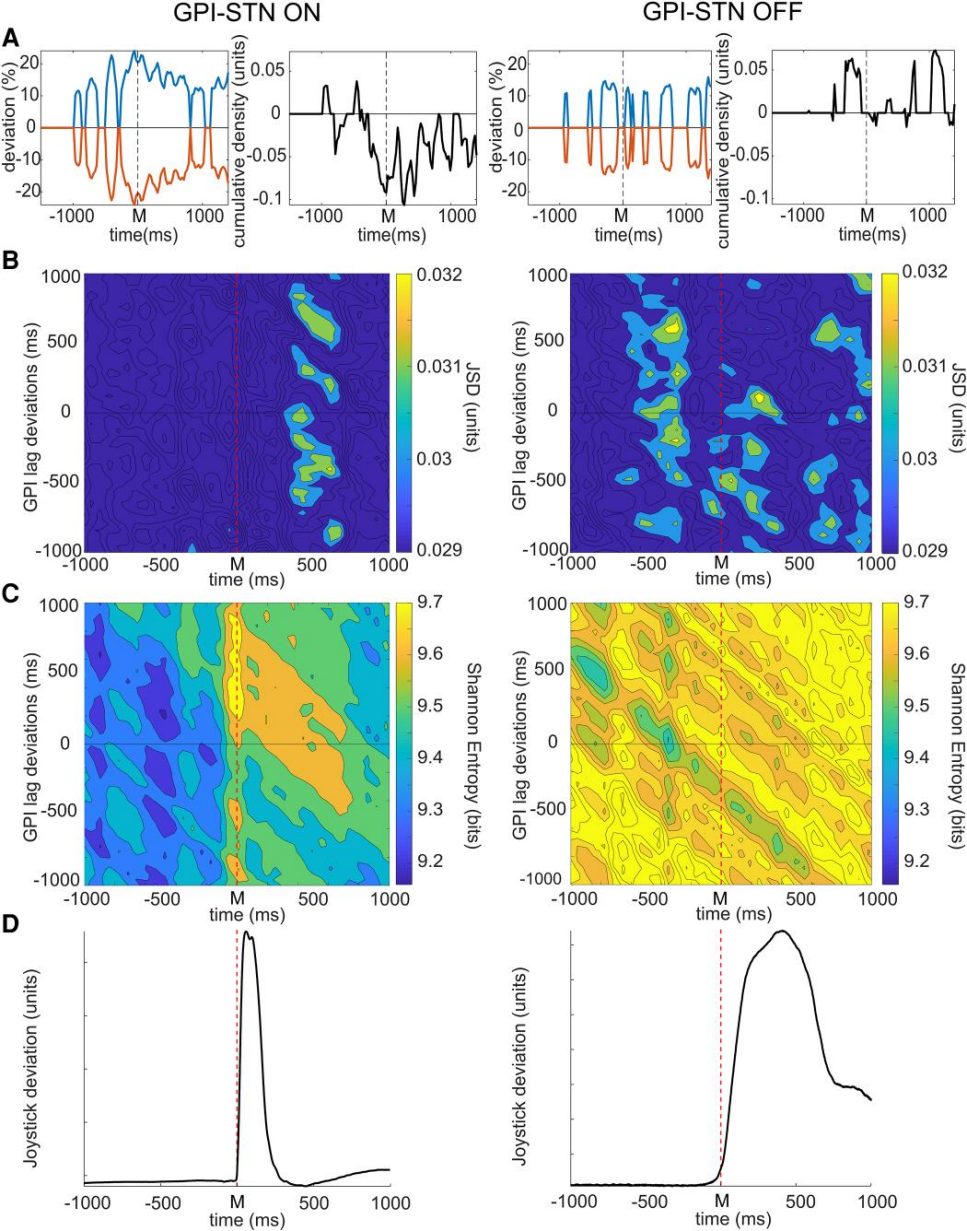
**Dopamine dependent GPI–STN network connectivity and entropy through the course of self-paced movement.** A. GPI–STN model deviations from baseline associated with spontaneous movements in pooled data (*n* = 26 on, *n* = 25 off, 2 subjects), left column images in treated (on) and right column in untreated (off) patients. Movements were undertaken at time 0, marked M. The blue upper lines represent the integrals of the positive differences and red lower lines, the negative. Treated patients show significantly greater degrees of model deviation (see supplemental). The neighbouring figures demonstrate differences between the maximal positive and negative deviations of the model from baseline in epochs with significant change. Model changes appear dominated by reductions in bivariate probability in treated patients, but vice versa in untreated patients. **B**. JSD of GPI–STN model with varying temporal relationship between the STN and GPI LFP activity. Maximal levels of interaction are demonstrated by thresholding to show the highest 30% of interaction levels. Increased functional connectivity is most evident in treated patients 300–675 ms after movements in a manner independent of specific GPI activity relationship (left column image, vertical band). In contrast untreated patients display similar levels of connectivity between the GPI and STN prior to and throughout movements (right column). **C**. Entropy in treated patients, with varied lagged relationship between GPI and STN activity shows an increase in entropy of activity between the regions at the time of movement (yellow indicative of higher levels, and blue lower). This is particularly for activity directed from STN to GPI (where STN activity leads GPI. In marked contrast significantly higher entropy is observed in untreated patients throughout the entire course of the trials independent of temporal relationship. **D**. Averaged joystick trajectory in treated (left) and untreated (right) patients. Statistical significance was derived via a two-tailed Wilcoxon rank sum test with a 0.05% significance limit for rejection of the null hypothesis as set out in the statistics section.

**Figure 2:**
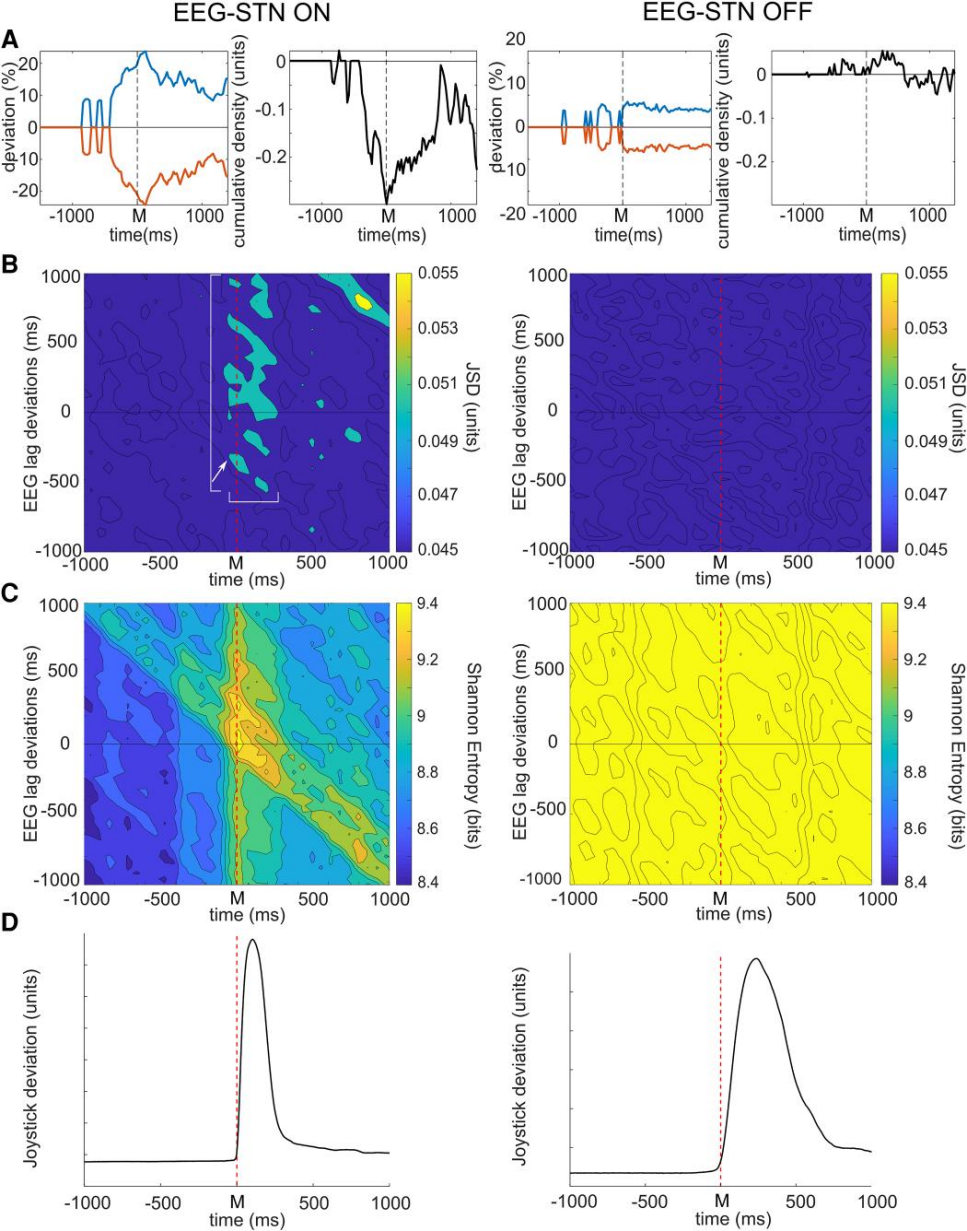
**Absence of dopaminergic treatment in the EEG–STN network is associated with loss of dynamic functional connectivity and high network entropy through the course of self-paced movement.** A. EEG–STN model deviations from baseline associated with spontaneous movements in pooled data (*n* = 57 on, *n* = 51 off, 4 subjects), left column images in treated (on) and right column in untreated (off) patients. Treated patients show significantly greater degrees of model deviation (see Supplementary material). The neighbouring figures demonstrate differences between the maximal positive and negative deviations of the model from baseline in epochs with significant change. Model changes appear dominated by reductions in bivariate probability in treated patients, but increase in untreated patients. **B**. JSD of EEG–STN model with varying temporal relationship between the STN and EEG LFP activity. As in Fig. 1 maximal levels of interaction are demonstrated by thresholding to show the highest 30% of interaction levels. Peaks in functional connectivity are seen between the two regions from 50 ms prior to 350 ms after movement (marked by the horizontal white band) with EEG activity that precedes the period by approximately 500 ms, up to at least 1 sec after movement onset (vertical white band). A non-lag dependent relationship is observed between STN activity and EEG activity otherwise (vertical band). An additional peak of interaction is noted for STN activity 800 ms after the movement with EEG activity a further 800 ms later. These patterns of connectivity relationship are lost entirely in the untreated state (right column). **C**. Entropy in treated patients, between EEG and STN shows an increase at the time of movement, particularly for activity directed from STN to cortex. Significantly higher entropy is observed in untreated patients throughout the entire course of movement independent of lag relationship (right column). **D**. Averaged joystick trajectory in treated (left) and untreated (right) patients. Statistical significance was derived via a two-tailed Wilcoxon rank sum test with a 0.05% significance limit for rejection of the null hypothesis as set out in the statistics section.

**Figure 3:**
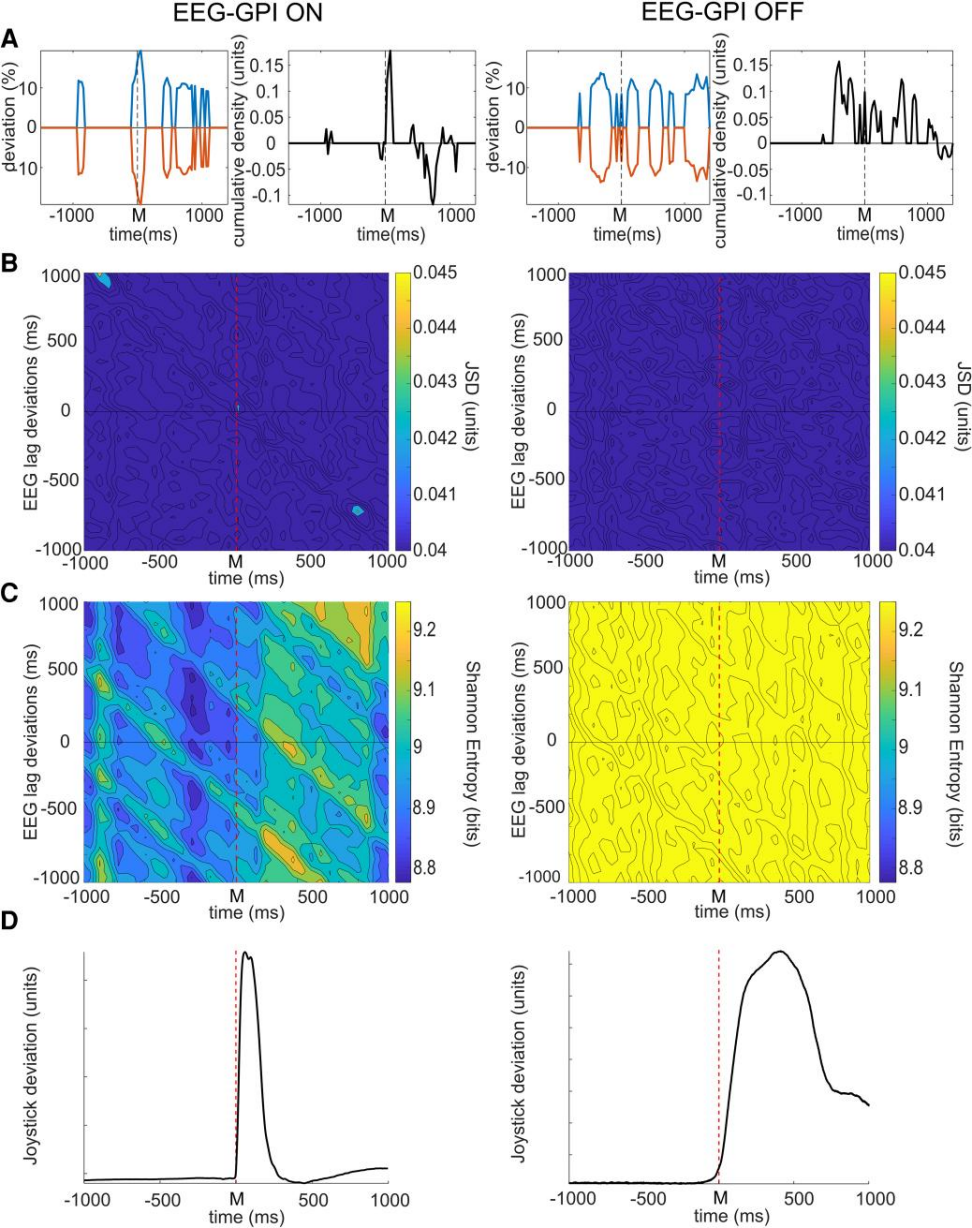
**Absence of dopaminergic treatment in the EEG–GPI network is associated with loss of dynamic functional connectivity and high entropy through the course of self-paced movement.** A. EEG–GPI model deviations from baseline associated with spontaneous movements in pooled data (*n* = 40 on, *n* = 35 off, 2 subjects), left column images in treated (on) and right column in untreated (off) patients, left column images in treated (on) and right column in untreated (off) patients. The neighbouring figures demonstrate differences between the maximal positive and negative deviations of the model from baseline in epochs with significant change. Model changes appear dominated by reductions in bivariate probability in treated patients but increases in untreated patients. **B**. JSD of EEG–GPI model with varying temporal relationship between the GPI and EEG LFP activity. As in Fig. 1 maximal levels of interaction are demonstrated by thresholding to show the highest 30% of interaction levels. In treated patients (left column) a significant increase in functional connectivity between GPI activity 850–950 ms prior to movement with EEG activity occurring at the time of movement is noted, implying directed interaction from GPI to cortex. A further peak of connectivity is evident between GPI activity 750–850 ms after the movement, with EEG activity at the time of movement onset (bottom right of image). There therefore appears to be reciprocal activity around the time of movement from GPI to cortex. This pattern of interaction is completely lost in the untreated state (right column). **C**. Entropy in treated patients, with lagged relationship between EEG and GPI activity does not show an increase in entropy at the time of movement but does show increased entropy for activity 375 ms after the movement with EEG activity approximately 450 ms prior to movement (see also Fig. 4C). In the untreated state, significant increase in entropy is noted throughout the course of motor trials independent of lag relationship (right column). **D**. Averaged joystick deviation in treated (left) and untreated (right) patients. Statistical significance was derived via a two-tailed Wilcoxon rank sum test with a 0.05% significance limit for rejection of the null hypothesis as set out in the statistics section.

The relationship between the LFP activities in two regions was modelled via development of a joint probability distribution of the field potential activity in the two sites. Such distributions were derived by generation of a bivariate kernel density estimate (kde) of activity using a Gaussian kernel.^22^ The cumulative density function (cdf) of the relationship was derived from this by multiplying the integral of the kde by a constant factor dependent on the resolution of the kde, such that the integral of the entire state space was 1. Models of the inter-regional relationship were thus derived for each trial considering 100 msec windows taken every 25 msec throughout the entire 3 sec of the movement blocks in the self-paced protocol, and the entire 5.5 sec of warning and cue presentation blocks of the externally cued protocol. Average models across trials for each window were then derived generating an average model relationship throughout the course of the trial. In each trial the baseline cdf activity was calculated by averaging the cdf across consecutive windows in a baseline period 1.5 to 1 sec prior to self-paced movements, and 1.5 to 1 sec prior to warning cue presentations in the self-paced and cues movement paradigms respectively. This baseline activity for each trial was then subtracted from each window in the trial to calculate deviations of the inter-regional relationship from baseline; and the average deviation was then calculated across trials. The positive integral of the 2 dimensional deviations was then calculated in each window to derive a percentage deviation of the model in that window from baseline. Mean percentage deviation in the baseline period was calculated and significant deviation from this was assessed as 2.5 sd percentage change from the baseline. It is the significant +ve and -ve deviations which are then represented in the left components (A) of Figs 1–3. The neighbouring A figures represent the difference between the greatest average maximal positive deviation from baseline within the 2 dimensional average deviations in a given window across trials with the greatest average negative deviation. While data were pooled across patients this was only undertaken for specific inter-regional comparisons where relevant data existed, e.g. for EEG–GPI models all subject data with EEG and GPI recordings in the treated and untreated state were considered. Data used noted in the relevant figure text.

### Functional connectivity derivation

Change in the relationship of the LFP between regions during tasks, may merely be corollary, and actual interaction was evidenced by considering differences between the observed joint probability distribution between regions, and that predicted by the marginal distributions. Where there is complete independence between probability distributions, the derived joint probability distribution of the marginals and the observed joint probability distribution should be the same. The marginal distributions were themselves derived via kernel density estimation of each regional activity independently. This difference between observed and predicted distributions was formalized by consideration of the Kullback–Leibler divergence (Eq. 1) between these predicted and observed distributions, i.e. the mutual information (MI), and the related normalized variant, the Jensen–Shannon divergence (JSD). These are presented below in equations 2 and 3, respectively, where *p(x, y)* and *p(x*)*p(y)* are indicative of the observed and predicted probability distributions respectively of activity *x* from the first region and *y* from the second region.^23^ While both the JSD and MI are symmetrical measures, the former has the advantages of being bounded by 0 and 1, allowing more obvious comparison.^24^ The use of both measures in the assessment of non-model dependent functional connectivity and brain network dynamics has previously been published by various authours.^23,25,26^ Functional connectivity thus derived was considered in a dynamic manner by considering the connectivity between windows of activity in the two region of 100 ms duration, moved sequentially in 25 ms steps throughout the trial in a manner analogous to the generation of the evolving joint probability distribution. In this manner, estimates of the degree of functional connectivity in each 100 ms window every 25 ms throughout the trial were generated allowing assessment of the evolution of levels of connectivity between the two considered regions (at a given temporal relationship) through the course of each trial. Higher levels of both JSD and MI thus derived are associated with greater degrees of disparity between observed and predicted probability distributions, and therefore greater levels of interaction between the regions considered.


(1) $$D_{kl}(\;p(x,y)∥\;p(x)p(y))=-\sum_{y\in Y}\;\sum_{x\in \chi }\;p(x,y)log\left(\frac{\;p(x)p(y)}{\;p(x,y)}\right)$$

(2) $$\mathrm{MI}(x,y)=\sum_{y\in Y}\;\sum_{x\in \chi }\;p(x,y)\log\left(\frac{\;p(x,y)}{\;p(x)p(y)}\right)$$

(3) $$\begin{array}{rl} \mathrm{JSD}(x,y) & =\frac{1}{2}D_{kl}(\;p(x,y)∥m(xy))\\ & \;\;\;+\;\frac{1}{2}D_{kl}(\;p(x)p(y)∥m(xy)) \end{array}$$

Where


$$m(xy)=\frac{1}{2}(\;p(x,y)+p(x)p(y))$$

It is noted that modelling of the relationship in this manner allows consideration of the Shannon entropy of the system. While kernel density estimation allowed for generation of estimates of the underlying continuous density function of the network relationship, calculation was descretized at a resolution of 2^6^ units. This allowed for consideration of the network entropy using Claude Shannon’s original discrete entropy formulation (Eq. 3^27^) without resorting to the use of differential entropy formulations:


(4) $$H(x,y)=\;-\sum p(x,y)log_{2}(\;p(x,y))$$

In addition the entropy of EMG activity, *z* was calculated with the univariate formulation as follows:


(5) $$H(z)=\;-\sum p(z)log_{2}(\;p(z))$$

### Time-lagged/Directed functional connectivity derivation

While both the JSD and MI are symmetric measures, i.e. JSD(*p(x)*∥*p(y)*) = JSD(*p(y)*∥*p(x)*), causal interactions, time-lagged connectivity or *directed functional connectivity* was examined by considering variation in the degree of functional connectivity with varying temporal relationship between the two LFP sources. An implicit assumption in this method is the belief that observed connectivity may appear maximal between LFP at the temporal lag of interaction between them. This was performed by calculation of both the JSD and MI between the two sources at windows in which single site activity was sequentially temporally shifted relative to the second site activity by 25 ms ranging from for example GPI activity leading the STN by 1 sec to GPI activity succeeding the STN activity by 1 sec. In this manner, interactions could be inferred by observation of maximal levels of connectivity with LFP in site *x*, leading that in *y*, and an estimate of the associated temporal lag of that interaction could be derived. While inferences may be made from this technique, no direct causality can be proven as mutual inputs to both considered regions may result in apparent functional connectivity independent of any directed interaction. The output of the time-lagged functional connectivity analysis was examined in plots of averaged functional connectivity throughout the course of the protocol across subjects, but for each variation in temporal relationship between considered nodes. A 2D representation was generated for each state, treated and untreated in which the strength of connectivity (magnitude of JSD and MI) was indicated by colour intensity at any given point during the course of a trial. Data thus generated was contour plotted via the cftool of Matlab to aid visualization. Analysis was however only undertaken on the raw data.

### Cortico-subthalamic and Cortico-EMG entropy correlations with EMG entropy

Probability distribution estimates of the standardized EMG activity were generated via kernel density estimation of successive 100 ms windows of activity at 25 ms intervals throughout the course of movement in each trial, in a manner analogous to the above described bivariate network interaction model derivation process. Activity 1 sec prior to 1 sec post movement was considered. EMG entropy was then derived for each 100 ms window with the discrete entropy formula above (Eq. 5). In order for correlation analysis of data across patients, entropy data for each subject was rescaled to range between 0 and 1. Correlation between network entropy and EMG entropy was derived via Pearson correlation coefficient calculation across trials considering 100 ms windows, as such correlation at time 0, the time of movement reflected entropy estimate correlations for activity from time 0 up to 200 ms after movement onset, reflecting activity during the joystick movement period.

### Statistical analysis

Differences between JSD, MI, and entropy in the treated versus untreated state were derived testing the null hypothesis that the median of the JSD/MI/entropy distribution in the treated state differs from that in the untreated state using a two-tailed Wilcoxon rank sum test with a 0.05% significance limit for rejection of the null hypothesis. The distribution in each state was derived by calculating the JSD/MI/entropy in each window in each trial and averaging across the trials to derive the average JSD/MI/entropy in a given window. Difference between the distributions in the treated and untreated state of the averaged JSD/MI/entropy across trials throughout the course of the period 1 sec prior to 1 sec after movement in the self paced, or 1.5 sec prior to warning cue to 1.5 sec after go cue in the cued paradigm (i.e. the periods depicted in the figures) were then tested as above. Correlation analysis between EEG–STN and EMG entropy was performed by calculation of the Pearson correlation coefficient between EEG–STN entropy in a given 100 ms windows across trials with the EMG entropy in the same window across trials. This was calculated in successive windows in 25 ms steps from 1 sec prior to 1 sec post movement onset and was examined separately in treated and untreated states. Analogous analysis was undertaken for EEG–EMG and EMG entropy correlation analysis.

### Data availability

The data that support the findings of this study are available from the corresponding author, upon reasonable request.

## Results

LFP, EEG, and EMG activity were analyzed across pooled data from seven PD patients in which either self-paced joystick movements or cued joystick movements after an initial warning cue were undertaken (Table 1) either on or off anti-PD medication. Across this data set this allowed for the consideration of network interactions between cortex and STN, cortex and GPI, and between GPI and STN throughout the course of self-initiated movement, and between GPI and STN for externally cued movement. While much work has previously been undertaken considering linear measures of interaction between the basal ganglia nuclei and basal ganglia nuclei and cortex, particularly in the frequency domain,^28–30^ the current investigation sought to limit preconceptions of the nature of interactions between the regions by adopting a method that does not assume linearity or oscillatory activity significance, modelling activity between areas in a single bivariate probability density via kernel density estimation. Joint probability distributions generated in this manner were observed to be non-stationary, demonstrating significant deviation from baseline levels of activity prior to either cues for movement or spontaneous movements, and such changes were often observable in the course of single trials. This phenomenon and details of changes in the modelled relationship between specific regions in the self-paced and cued paradigms are documented in the supplemental information.

Dynamic changes in the bivariate probability distributions could potentially result merely from changes in field potential activity in only one of the two regions being considered, and as such evidence of actual interaction between regions was sought. The levels of such interaction between regions of the network could be derived in a statistical manner by consideration of the difference between the observed activity relationship and that predicted by activity independently in each region. This was quantified by the JSD and MI, the former having the additional advantage of being bound by 0 (complete independence) and 1 (complete dependence). While interactions between simultaneously occurring activity in each region were considered initially, such a choice of relationship is essentially arbitrary and the relationships were considered in a systematic manner by analysis of interactions with varying temporal relationships of one area relative to another with up to 1000 ms difference. This approach also allowed levels of variability in each network interaction to be considered by derivation of the Shannon entropy of the modelled bivariate probability distributions.

### Self-paced movement network dynamics

#### GPI–STN interactions

Connectivity analysis showed that treated patients show little evidence of alteration in degrees of interaction between the two nuclei prior to or during movement (note average movement trajectory in Fig. 1D, however, a significant increase in connectivity occurs approximately 300 to 675 ms after movement occurrence (Figs 1B left and 4A). Lag analysis between the two sites is represented in Fig. 1B. Deviations on the y axis indicate the degree to which GPI activity leads (-ve) or succeeds (+ve) STN activity at a given time on the *x* axis. Higher levels of interaction between regions are indicated in yellow and lower in blue. This demonstrated that the increase in functional connectivity after movement occurs independent of specific lag of the GPI relative to the STN, implying that during this period the STN activity becomes more ‘GPI-like’. In contrast, off medication significant increase in functional connectivity between the two nuclei occurs prior to and during the movement (Figs 1B right and 4A).

**Figure 4:**
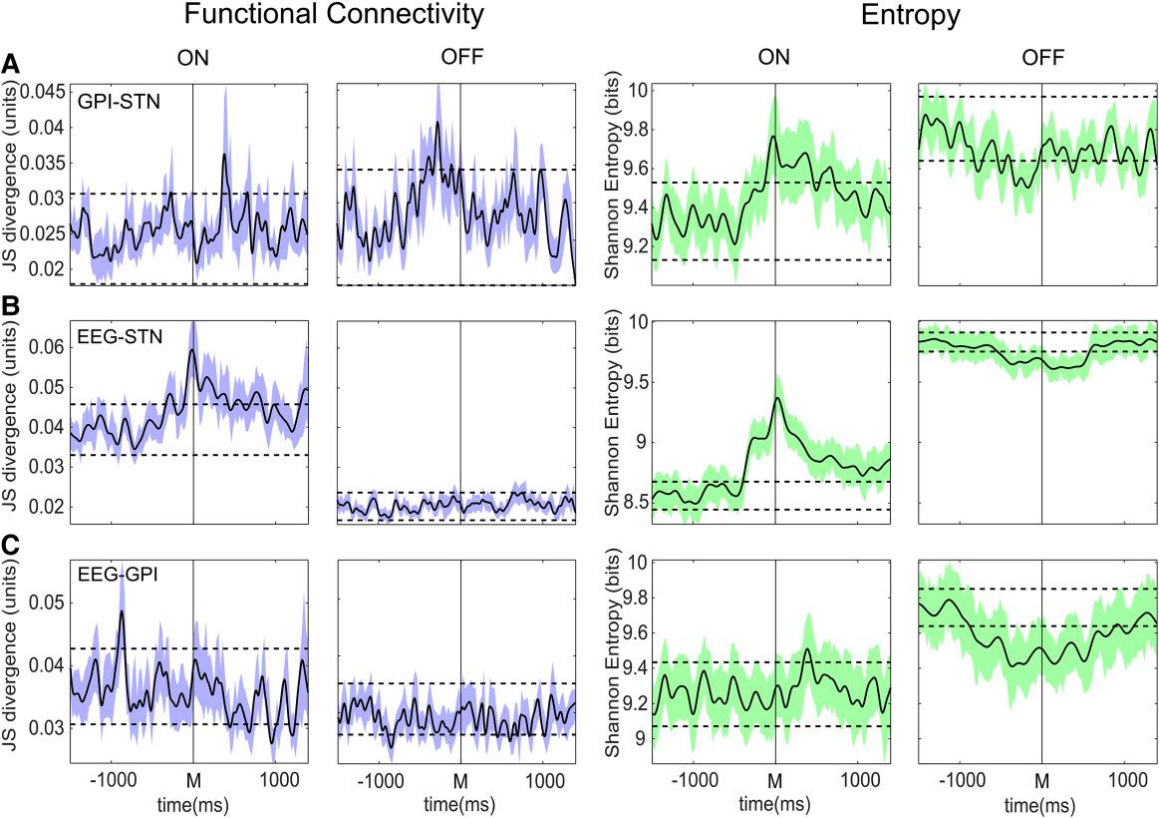
**Functional connectivity and entropy network relationships with specific temporal relationships between regions.** A. Interactions between GPI and STN in treated (ON) and untreated (OFF) subjects. JSD (blue, left 2 columns) and entropy (green, right 2 columns) with STN leading by 450 ms in treated and with GPI leading by 150 ms in untreated patients. Standard errors of the mean activity are represented by the respective colour margins either side of the mean (black line). Dotted lines indicate 2.5 sd significance levels. **B**. Interactions between EEG and STN in ON and OFF subjects. JSD (blue, left 2 columns), and entropy (green, right 2 columns) with EEG lagging the STN activity by 250 ms in treated patients (on), and with synchronous activity in untreated (off) patients. Significant modulations in connectivity from baseline are demonstrated in the treated patients which are absent in the untreated. In addition, markedly higher levels of entropy are noted between the untreated and treated states. **C**. JSD (blue, left 2 columns), with EEG lagging GPI activity by 975 ms in treated patients and entropy (green, right 2 columns) with EEG leading GPI by 875 ms in treated patients, and with synchronous activity in untreated patients (rightmost column). The significant early peak in functional connectivity is demonstrated, and the lower level of connectivity in the untreated state is evident.

Analysis of entropy in the network interaction between GPI and STN in treated patients demonstrated a significant increase in entropy from baseline, peaking at the time of movement onset (Figs 1C left and 4A). This was dominated by interactions between STN activity at the time of movement directed towards GPI activity between 200 and 800–900 ms later. It is of note that this did not coincide with the observed period of maximal functional connectivity between the two regions. In contrast, in untreated patients, the entropy in the inter-regional interaction was dominated by a significant increase relative to that in treated patients throughout the trials (*P* < 0.0001, mean 9.66 versus 9.45 bits, Figs 1C right and 4A). In addition, a reduction in entropy was observed around the time of movement initiation (Fig. 4A).

### Cortico-STN and Cortico-GPI interactions

Temporal patterns of connectivity between cortex and STN, and cortex and GPI were observed to differ through the course of movement in the treated state. The relationship between cortex and STN in treated patients demonstrates directed functional connectivity from EEG to STN appearing approximately 350 ms prior to movement (Fig. 2B left). EEG activity at this time is observed to interact with STN activity occurring approximately 50 ms prior to 350 ms after movement onset (Fig. 2B, horizontal white band). STN activity within this time window also shows a non-lag dependent relationship with EEG activity up to 1 sec after movement (Fig. 2B vertical white band), suggesting that either the STN activity in this period becomes ‘EEG like’ or that reciprocal directed connectivity from the STN to the EEG occurs (Fig. 2B, left). The earliest interaction appears to be for EEG activity approximately 350 ms prior to movement with STN activity 50 ms prior to movement (white arrow), implying initial directed interaction from EEG to STN at this time. In contrast, the relationship between EEG and GPI in treated patients demonstrates initial significantly increased connectivity between GPI activity occurring approximately 850 to 950 ms prior to movement with activity occurring in the EEG approximately 950 ms later, i.e. at the time of movement, implying directed interaction from the GPI to cortex (Fig. 3B left and 4C). A further peak of interaction is then observed approximately 800 ms after movement between GPI activity at that point and EEG activity preceding it by approximately 700 ms, i.e. around the time of spontaneous movement, implying reciprocal directed interaction from cortex back to GPI. These patterns of interaction between EEG and the LFP of both regions are lost completely in the untreated state (Figs 2B and 3B, right columns; EEG–GPI *P* < 0.0001, mean untreated versus treated JSD 0.029 versus 0.036; *P* < 0.0001 mean untreated versus treated MI 0.12 versus 0.15; EEG–STN *P* < 0.0001, mean untreated versus treated JSD 0.024 versus 0.044; *P* < 0.0001, mean untreated versus treated MI 0.10 versus 0.20).

Patterns of entropy also are observed to differ in the EEG–GPI and EEG–STN interactions between network links and between treated and untreated patient states. The treated EEG–STN interaction similar to the GPI–STN interaction demonstrates a significant increase in variability of the linked activity just prior to and at the onset of movement (Figs 2C left and 3B). This appears dominated by STN activity at the time of movement onset, leading the EEG by up to 500 ms. In contrast, while EEG–GPI interaction again shows an increase, this is apparent about 375 ms after movement for activity in the EEG approximately 450 ms prior to movement. Most obviously, in both network interactions, however, entropy in the untreated condition is dominated by significantly greater levels independent of any lag relationship and with reduction in entropy occurring prior to and during movement (Figs 2C and 3C, right, Fig. 4B and C); (EEG–STN *P* < 0.0001, untreated versus treated mean 9.75 versus 8.80 bits); (EEG–GPI *P* < 0.0001, untreated versus treated mean 9.58 versus 9.27 bits).

### Externally cued movement network dynamics

#### GPI–STN interactions

To clarify if the functional connectivity and entropy patterns of network connections described above were specific to the generation of movement itself, data from an externally cued protocol was also considered. In this paradigm, the subjects again performed a joystick movement, however, this was performed after presentation of a ‘go’ cue. This action cue was itself preceded by a warning cue, allowing for motor preparation which occurred 2.5 sec before the go cue itself (see methods). Significant digression of the modelled relationship between STN and GPI occurred after *both* warning cue and go cue presentation in the treated state (further detail available in the Supplementary material).

**Figure 5:**
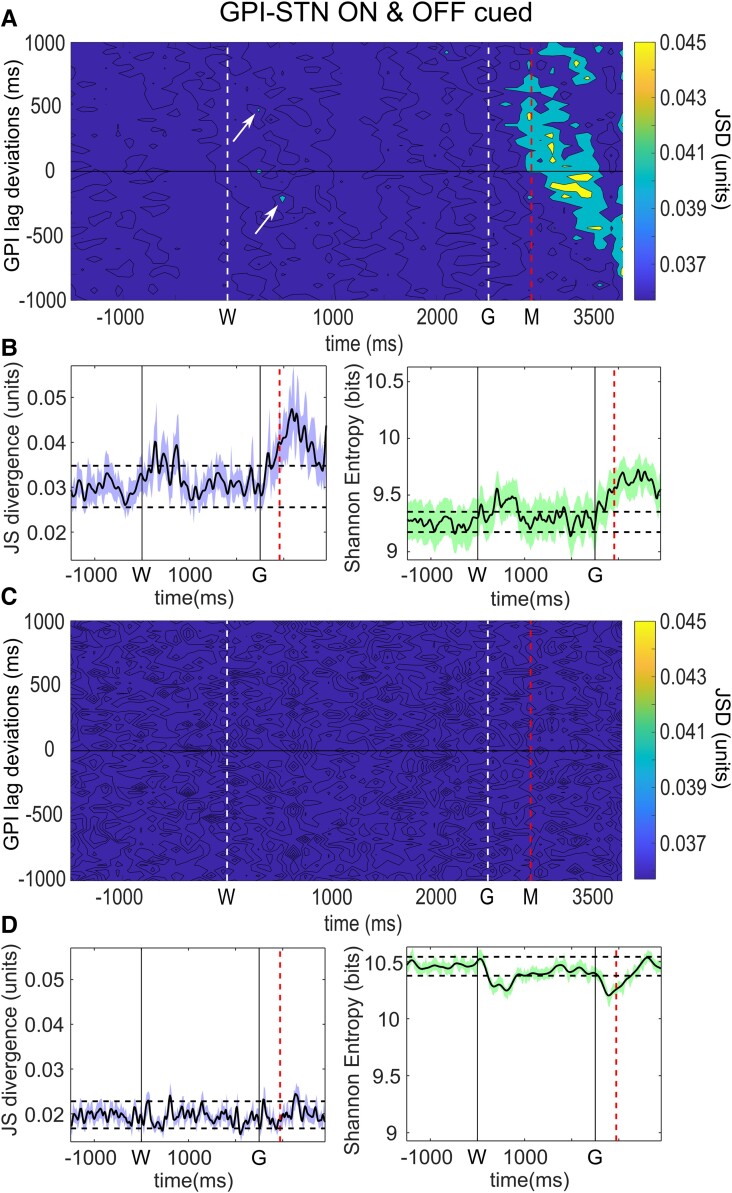
**Absence of dopaminergic treatment in the GPI–STN network is associated with loss of increases in functional connectivity associated with movement related cue presentation and high entropy levels.** A. JSD of GPI–STN model with varying temporal relationship between the STN and GPI LFP activity in treated subjects (*n* = 77 treated, 2 subjects). As in Fig. 1 maximal levels of interaction are demonstrated by thresholding to show the highest 30% of interaction levels. Significant peaks of functional connectivity are noted after both warning cue and go cue presentations. After the warning cue, 3 peaks are noted, a zero-lag peak possibly secondary to a common time locked input to both nuclei; directed functional connectivity from STN to GPI leading by 500 ms (upper white arrow); and reciprocal activity from GPI to STN with GPI activity leading the STN by 200 ms (lower white arrow). After the go cue, a more complex pattern is observed dominated by increased functional connectivity after the mean reaction time (**M**). **B**. JSD (blue, left image) and entropy (green, right image) with STN and GPI synchronous activity in treated patients. A significant increase in both is noted after both warning and go cues (dotted line indicates 2.5 sd from baseline mean). **C**. JSD of GPI–STN model with varying time lags in untreated patients (*n* = 57 untreated, 2 subjects). No significant cue locked interactions are noted. **D**. JSD (blue, left image) and entropy (green, right image) with STN and GPI synchronous activity in untreated patients. The interactions noted in the treated state are absent, mean baseline levels of connectivity are noted to be significantly lower, and entropy levels as in self-paced movements are noted to be significantly higher. Statistical significance was derived via a two-tailed Wilcoxon rank sum test with a 0.05% significance limit for rejection of the null hypothesis as set out in the statistics section.

In treated patients, significant increases in functional connectivity between GPI and STN were observed to occur after both go cues *and* warning cues (Fig. 5A and B left), seen initially within 150–250 ms of each cue. As such, increases in functional connectivity were not specific to motor activity itself but also observed after motor related cues. Analogous to the observations of model deviation, the magnitude of the go cue coupling between GPI and STN was significantly greater than that observed with the warning cue (*P* < 0.001 for JSD mean significant within 1 sec 0.034 versus 0.04 and *P* < 0.0001 MI mean 9.39 versus 9.57). Review of the lag analysis further suggested a pattern of interaction after the warning cue of initial synchronous connectivity between the nuclei, and at the same time directed information from STN to GPI with a 500 ms lag, and at the same time from GPI to STN with a 200 ms lag. After the go cue, a more complex pattern of interaction was apparent, similar to observed interactions in the self-paced task this again appeared dominated by interactions independent of GPI lag, and predominantly occurred after the mean reaction time. In untreated patients, baseline levels of connectivity were again significantly lower than in treated (*P* < 0.0001, treated JSD mean 0.030 versus untreated 0.020, treated *P* < 0.0001 MI treated 0.131 versus untreated 0.087 bits), and little evidence of significant increase in the level of interaction was observed after either cue (Fig. 5C and D left).

### Cortico-basal ganglia network relationship to cortical output

**Figure 6:**
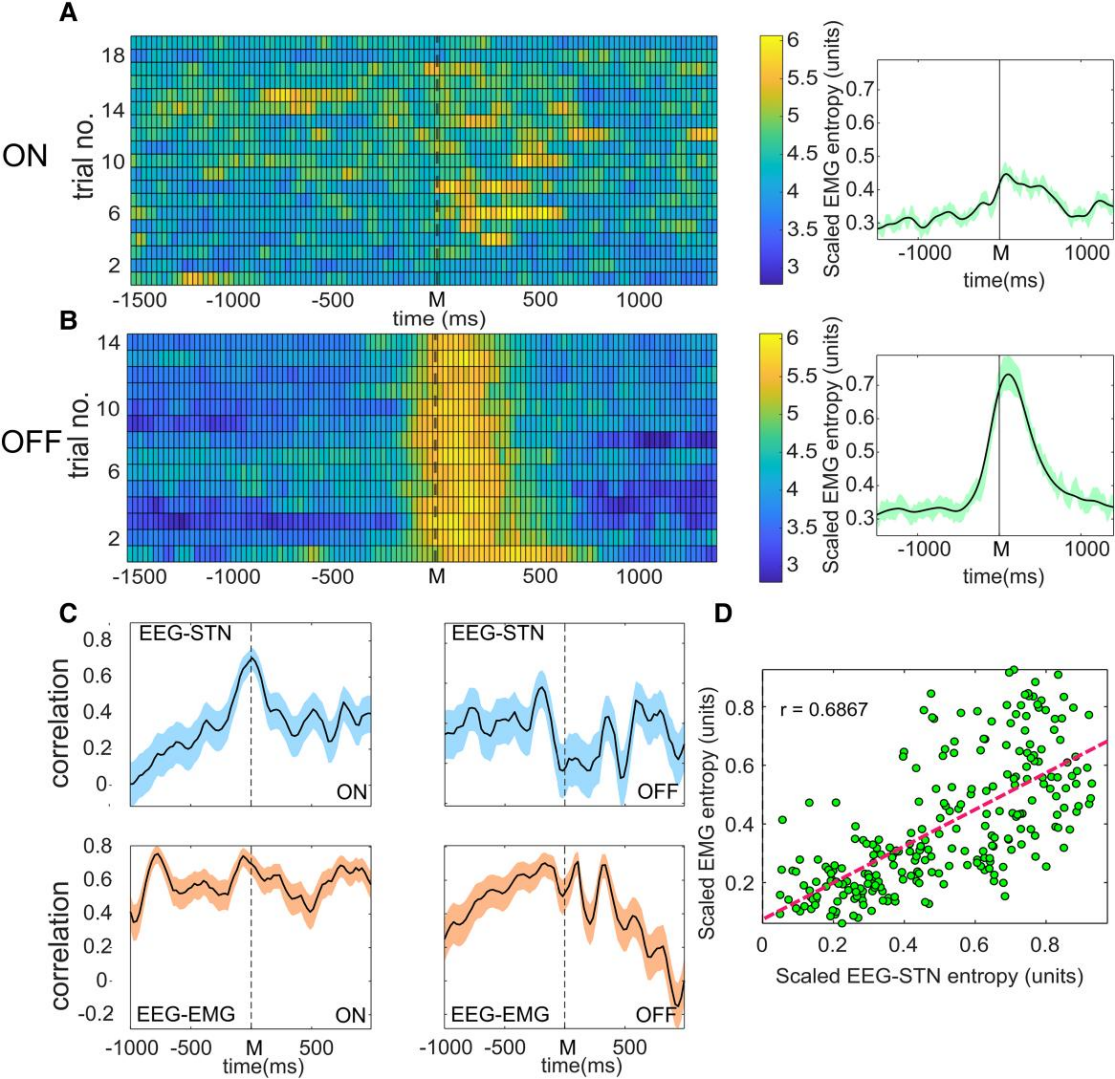
**The relationship between Parkinson’s patients’ EMG entropy and cortico-basal ganglia entropy.** A. Entropy levels throughout the course of successive self-paced movements in subject 4 on treatment, and B off treatment. Movements were undertaken at time 0, marked M. When off treatment increased muscle entropy with movement is observed consistent with PD EMG abnormalities. Average scaled entropy across trials 1500 msec either side of movements in treated and untreated states are indicated to the right of the trials (treated *n* = 51, untreated *n* = 40, 3 subjects; standard errors of the means are indicated either side of the mean line). **C**. Correlation between EEG–STN (top figures), and EEG–EMG (bottom figures) network entropy and EMG entropy through the course of self-paced movement. 95% confidence limits are indicated either side of the correlation. Significant correlation is noted between cortico-basal ganglia (EEG–STN) entropy and EMG entropy in the treated patient state (ON) at the time of movement, which is significantly reduced in the untreated state (OFF). In contrast, significant EEG–EMG correlation with EMG entropy is observed irrespective of treatment state. **D**. EEG–STN entropy correlation with EMG entropy at the time of movement observed in C ON (*P* < 0.0001). Correlations were assessed using the Pearson correlation coefficient as set out in the statistics section.

In an effort to clarify how the dynamic connectivity and entropy changes described above relate to cortical outputs, and the actual symptoms of PD themselves, network relationships to EMG activity around the time of movement were considered. Variability in features such as EMG burst duration, and the probability of short first agonist EMG bursts, are established phenomena,^31–33^ and evidence of irregularity of the PD patient EMG. As such entropy or variability of the EMG itself was reviewed. Averaged EMG entropy through the course of movements showed elevation peaking at the time of movement, which was also visible on a trial by trial basis (Fig. 6A and B). Consistent with expectations, the degree of elevation in the observed EMG entropy was significantly greater during movement in the untreated than treated PD state, (*P* < 0.0001 mean scaled entropy 100 ms either side of movement treated 0.37 versus untreated 0.65).

Cortico-basal ganglia network activity variability demonstrated by EEG–STN entropy was observed to be significantly correlated with the degree of variability in the EMG in treated patients at the time of movement (*r* = 0.69, *P* < 0.0001, Pearson correlation coefficient, no of trials = 51 Fig. 6C and D), but this relationship was significantly weaker in the dopamine depleted untreated state during movement itself (*r* = 0.16, *P* = 0.02, Pearson correlation coefficient, no of trials = 40; see Fig. 6C). This appeared consistent with the apparent loss of functional interaction between the STN and GPI nuclei of the basal ganglia and cortex reported above. Furthermore, however, it appeared incompatible with a direct link between the variability of the EMG in untreated PD patients and cortico-basal ganglia connectivity, i.e. a ‘noisy signal’ generated by the basal ganglia themselves inducing motor irregularities. In contrast, examination of the EEG–EMG activity variability relationship with EMG entropy showed significant correlation with movement in both treated and untreated conditions (*P* < 0.0001 *r* = 0.71 versus *P* < 0.0001 *r* = 0.50, Pearson correlation coefficient), suggesting that any derived EMG variability was more related to cortical input distinct from the basal ganglia.

## Discussion

### Cortico-basal ganglia network entropy

The findings presented here are novel in demonstrating not only marked differences in the level of entropy or variability in cortico-basal ganglia and intra-basal ganglia connectivity with dopamine state in PD patients, but the temporal dynamics of this variability through the course of spontaneous movement. An ‘entropy window’ is observed to exist between treated and untreated states in which in the treated state network variability increases, and in the untreated it decreases. This occurs around the time of movement initiation, a period of presumed information transmission related to motor task. These observations fit within a growing body of work that has not only demonstrated abnormal patterns of neuronal activity in various regions of the basal ganglia in PD and animal PD models,^16,34–36^ but also suggested neuronal and population entropy changes^37–39^ and a potential mechanism of DBS in improving PD symptoms by lowering entropy.^37^ Beyond the observed dopamine dependence of cortico-basal ganglia entropy, correlation has also been demonstrated here between cortico-subthalamic network entropy and a marker of PD symptom pathology during movement, but only in treated patients. This latter observation appears in contrast to prior work that has shown correlation between gait freezing and STN entropy.^40^ That relationship was however observed solely in the untreated state without a comparison with treated patients, was not shown in actual network entropy but solely local nucleus entropy, and was only evident in one sub-frequency band of the analysis while absent in another. Similarly, recent evidence has been presented of correlation between STN LFP entropy and PD motor severity scores (motor Unified Parkinson’s disease Rating Scale) in untreated patients.^41^ Again, no treated patient data was available for comparison, only local LFP activity was considered (rather than network interaction), and the epochs considered did not allow for transient temporal changes to be evident as presented here. It is also noteworthy that correlation was not with any marker of motor activity during actual task performance and no correlation with tremor or rigidity scores was observed.^41^

### Cortico-basal ganglia functional connectivity

**Figure 7:**
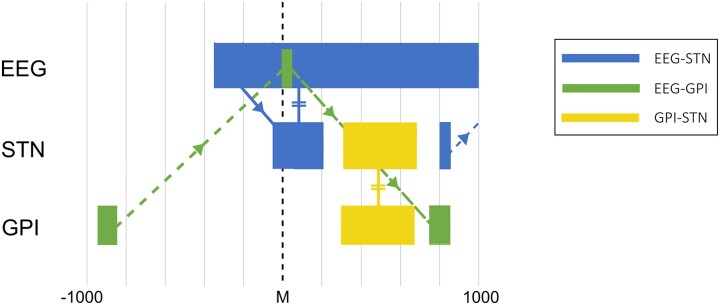
**Schematic representation of network interactions between the Cortex, STN, and GPI during the course of self-paced movements in treated PD patients.** EEG–STN, EEG–GPI, and GPI–STN interactions are represented by blue, green, and yellow regions of the plot. Onset of movement is indicated by M, at time 0. The arrows depict the direction of functional connectivity interactions, and the = signs where the STN takes on the character of the region it is interacting with. (GPI = globus pallidus interna, STN = subthalamic nucleus).

With regards the observed patterns of functional connectivity in the basal ganglia network presented here, the significant deficit in untreated patients relative to treated between cortex and the basal ganglia, supports several similar observations in fMRI of PD patients in both states at rest^42–44^; and of dopamine related frequency domain changes noted between basal ganglia and EMG^45^ and to some extent between GPI and STN^28^ during movement. The approach adopted here however has allowed characterization of both the network temporal features of changes in each state during movement and comparable assessment of their extent, which are illustrated schematically in Fig. 7 in treated patients.

If these patterns are considered individually, with regards cortico-pallidal functional connectivity, interaction is noted between activity in the GPI preceding the movement by hundreds of milliseconds and cortical activity at the time of movement. This may be considered in the context of evidence of neuronal basal ganglia activity (specifically in the striatum) observed in primate single unit recordings that precede self-initiated movement by as much as 2 sec.^46^ Specifically, in the pallidum, depth electrode recordings of pre-operative epileptic patients have recorded Bereitschaftspotential (or readiness potential) activity with onset of a similar timescale suggesting basal ganglia as well as cortical generation of this phenomenon.^47^

Here functional connectivity between cortex and STN with onset several hundred milliseconds before movement, and initiated from cortex to STN is reported. Substantial preceding work considering oscillatory LFP relationships between cortical and STN activity recorded via DBS electrodes in various patient populations has been published. Functional connectivity analyses in the resting state in PD patients both on and off medication have demonstrated significant alpha (7–13 Hz), beta (13–30 Hz) and high gamma band (70–80 Hz) linkage.^29,48–50^ Furthermore, at least in the alpha and beta bands, this resting state interaction has been deemed to primarily be directed from cortex to STN independent of dopamine state.^29,44,48^ While the current work does not presume linear oscillatory relationships, these may well be prominent components of the relationship here observed.

Concerning intra-basal ganglia GPI–STN functional connectivity, prior frequency domain analysis has shown evidence of an increase in oscillatory gamma frequency (specifically ∼70–80 Hz) coherence between nuclei in both cued and self-paced movement paradigms in treated PD patients^28^ not observed in untreated patients which may correspond to the increased functional connectivity noted in the network activity reported here. This likely does not reflect a phenomenon specific to PD, as local gamma frequency LFP oscillatory power increases with movement (with corresponding beta frequency reductions) have been reported in dystonic patients^51^ as well. Of note, both the oscillatory coupling and local frequency changes appear to occur predominantly subsequent to movement onset with similar timing to the connectivity changes seen here.

### A novel conception of the noisy signal hypothesis

While the above findings remain consistent with the hypothesis of basal ganglia noise induced abnormal information transmission underlying some of the symptom complex of PD, the lack of correlation between variability within the cortico-basal ganglia network and EMG in the untreated state observed here, appears contradictory.

**Figure 8:**
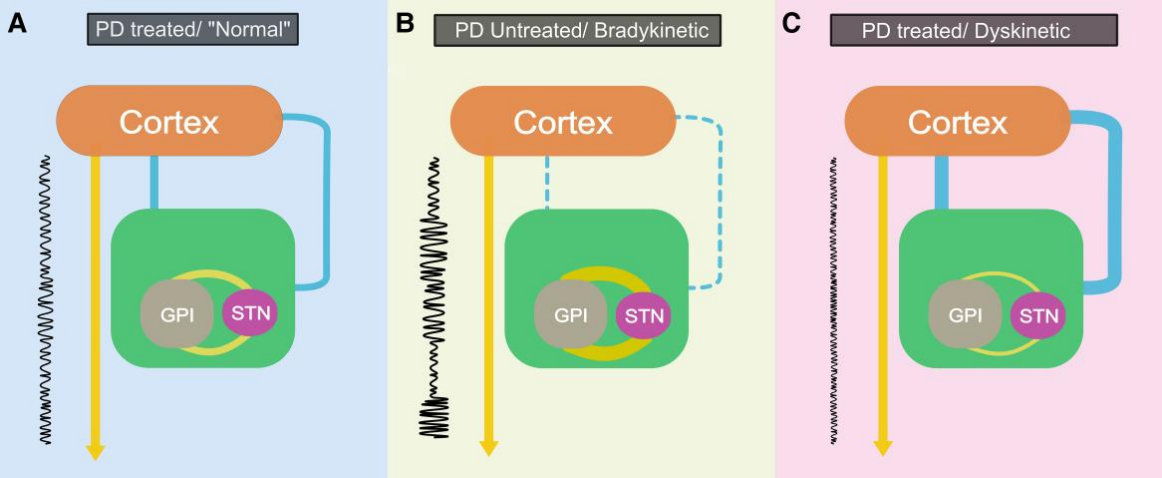
**A functional connectivity based model of the basal ganglia relationship to cortical outputs.** Connections depicted are representative of functional connectivity not direct anatomical links with the basal ganglia indicated by the green square. Connection thickness is indicative of connection strength. **A**. In the treated PD state, intra-basal ganglia interactions allow interaction of the basal ganglia with cortical inputs and are associated with levels of cortical output uncertainty (output arrow) consistent with optimal information transmission (indicated by the schematic ‘output’ signal to the left of the figure). **B**. In PD patients, off medication increased pathological intra-basal ganglia interactions (thick yellow line), and abnormal neuronal activity patterns impair effective functional interactions between the basal ganglia and cortical inputs and are associated with allowing high levels of cortical output uncertainty/variability. **C**. With chronic/excessive PD treatment high levels of basal ganglia cortical connectivity are induced associated with excessive ‘regularization’ of cortical outputs limiting the potential complexity of information transmission. (GPI = globus pallidus interna, STN = subthalamic nucleus).

It may therefore be helpful to consider a new version of the noisy signal hypothesis that attempts to reconcile the observations made to date. In this conception, intelligible information transmission of system signals is the primary model output (see Fig. 7), whether those be motor or cognitive domains relevant to the basal ganglia. In addition, functional connectivity in this model is considered to define important relationships between nuclei and regions, beyond direct anatomical connection (though at least indirect pathways for communication must exist). In the treated state significant functional connectivity exists between cortex and the nuclei of the basal ganglia, and whether by fault or design this interaction is associated with maintaining a degree of variability of network transmission consistent with the ability to transmit sufficiently complex information (Fig. 8A).

### Bradykinesia

This model suggests that in the untreated Parkinsonian state, pathological basal ganglia changes manifest at a neuronal scale as aberrant firing patterns, and at a population level as oscillatory beta synchrony in LFPs,^15–17,52^ are associated with altered intra-basal ganglia connectivity and significantly decreased effective cortico-basal ganglia connectivity. In the absence of cortico-basal ganglia interaction, higher levels of cortical output signal variability occur impairing the clarity of signal transmission (Fig. 8B). Recent modelling of cortical oscillatory relationships to the basal ganglia in specific frequency bands has suggested a similar gating role of the basal ganglia.^53^ In information theoretic terms, this may be considered analogous to the rate limitation of transmission in a noisy channel conceived by Claude Shannon, in which channel capacity is dependent upon the signal to noise ratio of the channel; and signal transmission at a rate greater than channel capacity may be associated with increasing probabilities of error transmission and with ‘…required delays at transmitter and receiver increasing indefinitely’.^54^ In the PD patient motor domain, this may be manifest as bradykinesia secondary to limitation of the rate at which correct motor information may be received.

### Dyskinesia

Dyskinesia in this model then results from excessively low levels of variability in cortical output, in the context of high levels of connectivity between basal ganglia and cortex (Fig. 8C). While information may be readily transmitted under such conditions, signal content may be insufficiently complex to subtly control the target muscle population. This would appear consistent not only with observations suggesting that neuronal activity changes seen in GPI with dopaminergic treatment in PD patients occur to a yet greater extent in PD patients with dyskinesia,^55,56^ but also observations of increased frequency domain connectivity between cortex and basal ganglia in the 6-OH DA rat model of PD,^57^ and particularly high degrees of gamma oscillatory activity (associated with the treated Parkinsonian state) in dyskinetic medicated patients.^58^

### Dystonia

An entropy based model has already been proposed by Darbin *et al*.^59,60^ in which dystonia is considered a hyperkinetic state associated with low basal ganglia entropy. The observed reduction in functional connectivity of the basal ganglia to sensorimotor cortex in dystonia^61,62^ in the context of the current model may however suggest that as in the case of Parkinsonian bradykinesia aberrant levels of variability in associated networks may underly the motor symptoms of dystonia. The distinction in the exact motor manifestations expressed from PD, may be explained by differences in the network (or ‘domain’) in which abnormal activity is permitted. In dystonia, this may well be cerebello–thalamo–striatal pathways.^63^

## Limitations

The requirements of human post-operative recordings were a limitation in the present study, necessitating LFP acquisition without associated unit activity. LFP activity as presented here are considered indicative of summative potential fluctuations at synapses and other subthreshold membrane fluctuations and are as such considered indicative of integrative activity underlying action potentials. Variability measures as presented are therefore not direct measures of information but reflect variability of processes associated with information transmission. It is also noted that signal to noise ratio fluctuation in dynamic time series may potentially result in apparent alterations in connectivity; however, this would not appear to be consistent with the disparity between entropy and connectivity fluctuations observed here, and would not appear to explain the state related disparities. In addition, post DBS electrode insertion ‘stun effect’ may potentially result in symptom improvement, but this again would not appear to differ in any effect upon treated and untreated states if present. Finally, given the small data set available for the current analysis and potential inter-subject variability, a degree of caution is required in the interpretation of the specific temporal patterns of functional connectivity noted. The broader observations of marked reductions in functional connectivity between cortex and basal ganglia with dopamine state, and marked increases in network entropy throughout the network in untreated patients appear to be the most robust findings.

## Conclusions

Do the findings and derived model presented here help to resolve the paradoxes of current conceptions of basal ganglia function? Well, with regards the benefit of pallidotomy and GPI DBS on both ‘hyperkinetic’ and ‘hypokinetic’ disorders, as in the prior noisy signal hypothesis this may be explained by the dependence of symptom improvement with such interventions ultimately upon improvements in effective information transmission. Indeed the current model may explain why both subthalamotomy which may be expected to decrease GPI output neuron rates and STN DBS which has actually been observed to *increase* GPI neuronal firing may both be of benefit in PD.^64^ Ultimately, all of these changes may be associated with alterations in firing patterns that improve effective functional connectivity with regions beyond the basal ganglia which may in turn be associated with improvement in uncertainty in information transmission related to regionally associated networks. The ability of abnormal patterns of activity and oscillatory synchrony to produce disparate symptoms may similarly be explained as has been above. While many questions remain the model presented here suggests that further insight concerning the means by which effective cortico-basal ganglia interaction are mediated, and mechanisms associated with ‘regularizing’ of basal ganglia neuronal activity patterns may be of great significance in advancing therapeutic interventions in PD and potentially other basal ganglia functional connectivity disorders.

## Supplementary material

Supplementary material is available at *Brain Communications* online.

## Supplementary Material

fcad123_Supplementary_Data

## Abbreviations

cdf =: cumulative density function
GPI =: globus pallidus interna
GPE =: globus pallidus externa
JSD =: Jensen–Shannon divergence
kde =: kernel density estimate
LED =: light-emitting diode
LFP =: local field potential
MI =: mutual information
STN =: subthalamic nucleus
SNc =: substantia nigra pars compacta
SNr =: substantia nigra pars reticulata
UPDRS =: Unified Parkinson’s disease Rating Scale

## References

[1] Smeets WJ , Marín O, González A. Evolution of the basal ganglia: New perspectives through a comparative approach. J Anat. 2000;196(Pt 4):501–517. 10923983 10.1046/j.1469-7580.2000.19640501.x PMC1468093

[2] Grillner S , Robertson B, Stephenson-Jones M. The evolutionary origin of the vertebrate basal ganglia and its role in action selection. J Physiol. 2013;591(22):5425–5431. 23318875 10.1113/jphysiol.2012.246660 PMC3853485

[3] Grillner S , Robertson B. The basal ganglia over 500 million years. Curr Biol. 2016;26(20):R1088–R1100. 27780050 10.1016/j.cub.2016.06.041

[4] Rogers FT . The functional significance of the extra-pyramidal systems. Psychol Bull. 1927;24(4):216–239.

[5] Bhatia KP , Marsden CD. The behavioural and motor consequences of focal lesions of the basal ganglia in man. Brain. 1994;117(Pt 4):859–876. 7922471 10.1093/brain/117.4.859

[6] Dickson DW . Parkinson’s disease and parkinsonism: Neuropathology. Cold Spring Harb Perspect Med. 2012;2(8):a009258. 10.1101/cshperspect.a009258 PMC3405828 22908195

[7] Albin RL , Young AB, Penney JB. The functional anatomy of basal ganglia disorders. Trends Neurosci. 1989;12(10):366–375. 2479133 10.1016/0166-2236(89)90074-x

[8] Penney JB Jr , Young AB. Striatal inhomogeneities and basal ganglia function. Mov Disord. 1986; 1(1):3–15. 2848190 10.1002/mds.870010102

[9] Nelson AB , Kreitzer AC. Reassessing models of basal ganglia function and dysfunction. Annu Rev Neurosci. 2014;37:117–135. 25032493 10.1146/annurev-neuro-071013-013916 PMC4416475

[10] Hariz M , Tabrizi S. Patients with Huntington’s disease pioneered human stereotactic neurosurgery 70 years ago. Brain. 2017;140(9):2516–2519. 29050388 10.1093/brain/awx193

[11] Wojtecki L , Groiss SJ, Hartmann CJ, et al Deep brain stimulation in Huntington’s disease-preliminary evidence on pathophysiology, efficacy and safety. Brain Sci. 2016;6(3):38. 27589813 10.3390/brainsci6030038 PMC5039467

[12] Liu Y , Li F, Luo H, et al Improvement of deep brain stimulation in Dyskinesia in Parkinson’s disease: A meta-analysis. Front Neurol. 2019;10:151. 30858823 10.3389/fneur.2019.00151 PMC6397831

[13] Marsden CD , Obeso JA. The functions of the basal ganglia and the paradox of stereotaxic surgery in Parkinson’s disease. Brain. 1994;117(Pt 4):877–897. 7922472 10.1093/brain/117.4.877

[14] Brown P , Eusebio A. Paradoxes of functional neurosurgery: Clues from basal ganglia recordings. Mov Disord. 2008;23(1):12–20. 17999423 10.1002/mds.21796

[15] Nini A , Feingold A, Slovin H, Bergman H. Neurons in the globus pallidus do not show correlated activity in the normal monkey, but phase-locked oscillations appear in the MPTP model of parkinsonism. J Neurophysiol. 1995;74(4):1800–1805. 8989416 10.1152/jn.1995.74.4.1800

[16] Bergman H , Wichmann T, Karmon B, DeLong MR. The primate subthalamic nucleus. II. Neuronal activity in the MPTP model of parkinsonism. J Neurophysiol. 1994;72(2):507–520. 7983515 10.1152/jn.1994.72.2.507

[17] Brown P , Oliviero A, Mazzone P, Insola A, Tonali P, Di Lazzaro V. Dopamine dependency of oscillations between subthalamic nucleus and pallidum in Parkinson’s disease. J Neurosci. 2001;21(3):1033–1038. 11157088 10.1523/JNEUROSCI.21-03-01033.2001 PMC6762327

[18] Brown P . Oscillatory nature of human basal ganglia activity: Relationship to the pathophysiology of Parkinson’s disease. Mov Disord. 2003;18(4):357–363. 12671940 10.1002/mds.10358

[19] Weinberger M , Mahant N, Hutchison WD, et al Beta oscillatory activity in the subthalamic nucleus and its relation to dopaminergic response in Parkinson’s disease. J Neurophysiol. 2006; 96(6):3248–56. 17005611 10.1152/jn.00697.2006

[20] Devergnas A , Pittard D, Bliwise D, Wichmann T. Relationship between oscillatory activity in the cortico-basal ganglia network and parkinsonism in MPTP-treated monkeys. Neurobiol Dis. 2014;68:156–166. 24768805 10.1016/j.nbd.2014.04.004 PMC4275129

[21] Swan CB , Schulte DJ, Brocker DT, Grill WM. Beta frequency oscillations in the subthalamic nucleus are not sufficient for the development of symptoms of Parkinsonian bradykinesia/akinesia in rats. eneuro. 2019;6(5). 10.1523/ENEURO.0089-19.2019 PMC6817717 31540998

[22] Botev Z , Grotowski J, Kroese D. Kernel density estimation via diffusion. Ann Stat. 2010;38(5):2916–2957.

[23] Mohanty R , Sethares WA, Nair VA, Prabhakaran V. Rethinking measures of functional connectivity via feature extraction. Sci Rep. 2020;10(1):1298. 31992762 10.1038/s41598-020-57915-w PMC6987226

[24] Lin J . Divergence measures based on the Shannon entropy. IEEE Trans Inform Theory. 1991;37(1):145–151.

[25] Amico E , Arenas A, Goñi J. Centralized and distributed cognitive task processing in the human connectome. Netw Neurosci. 2019;3(2):455–474. 30793091 10.1162/netn_a_00072 PMC6370483

[26] Wang Z , Alahmadi A, Zhu D, Li T. Brain functional connectivity analysis using mutual information. In: 2015 IEEE Global Conference on Signal and Information Processing (GlobalSIP). Orlando, FL, 2015:542–546. doi: 10.1109/GlobalSIP.2015.7418254.

[27] Shannon CE . The mathematical theory of Communication. 1963. MD Comput. 1997;14(4):306–317. 9230594

[28] Cassidy M , Mazzone P, Oliviero A, et al Movement-related changes in synchronization in the human basal ganglia. Brain. 2002;125(Pt 6):1235–1246. 12023312 10.1093/brain/awf135

[29] Williams D , Tijssen M, Van Bruggen G, et al Dopamine-dependent changes in the functional connectivity between basal ganglia and cerebral cortex in humans. Brain. 2002;125(Pt 7):1558–1569. 12077005 10.1093/brain/awf156

[30] Neumann WJ , Jha A, Bock A, et al Cortico-pallidal oscillatory connectivity in patients with dystonia. Brain. 2015;138(Pt 7):1894–1906. 25935723 10.1093/brain/awv109

[31] Berardelli A , Rothwell JC, Thompson PD, Hallett M. Pathophysiology of bradykinesia in Parkinson’s disease. Brain. 2001;124(11):2131–2146. 11673316 10.1093/brain/124.11.2131

[32] Hallett M , Khoshbin S. A physiological mechanism of bradykinesia. Brain. 1980;103(2):301–314. 7397480 10.1093/brain/103.2.301

[33] Robichaud JA , Pfann KD, Leurgans S, Vaillancourt DE, Comella CL, Corcos DM. Variability of EMG patterns: A potential neurophysiological marker of Parkinson’s disease? Clin Neurophysiol. 2009;120(2):390–397. 19084473 10.1016/j.clinph.2008.10.015 PMC2679966

[34] Filion M , Tremblay L, Bédard PJ. Abnormal influences of passive limb movement on the activity of globus pallidus neurons in parkinsonian monkeys. Brain Res. 1988;444(1):165–176. 3359286 10.1016/0006-8993(88)90924-9

[35] Hutchison WD , Lozano AM, Davis KD, Saint-Cyr JA, Lang AE, Dostrovsky JO. Differential neuronal activity in segments of globus pallidus in Parkinson’s disease patients. Neuroreport. 1994;5(12):1533–1537. 7948856 10.1097/00001756-199407000-00031

[36] Magnin M , Morel A, Jeanmonod D. Single-unit analysis of the pallidum, thalamus and subthalamic nucleus in parkinsonian patients. Neuroscience. 2000;96(3):549–564. 10717435 10.1016/s0306-4522(99)00583-7

[37] Dorval AD , Russo GS, Hashimoto T, Xu W, Grill WM, Vitek JL. Deep brain stimulation reduces neuronal entropy in the MPTP-primate model of Parkinson’s disease. J Neurophysiol. 2008;100(5):2807–2818. 18784271 10.1152/jn.90763.2008 PMC2585386

[38] Dorval AD , Muralidharan A, Jensen AL, Baker KB, Vitek JL. Information in pallidal neurons increases with parkinsonian severity. Parkinsonism Relat Disord. 2015;21(11):1355–1361. 26433544 10.1016/j.parkreldis.2015.09.045 PMC4631644

[39] Lafreniere-Roula M , Darbin O, Hutchison WD, Wichmann T, Lozano AM, Dostrovsky JO. Apomorphine reduces subthalamic neuronal entropy in parkinsonian patients. Exp Neurol. 2010;225(2):455–458. 20659454 10.1016/j.expneurol.2010.07.016 PMC2939318

[40] Syrkin-Nikolau J , Koop MM, Prieto T, et al Subthalamic neural entropy is a feature of freezing of gait in freely moving people with Parkinson’s disease. Neurobiol Dis. 2017;108:288–297. 28890315 10.1016/j.nbd.2017.09.002 PMC6386531

[41] Weber I , Florin E, von Papen M, Visser-Vandewalle V, Timmermann L. Characterization of information processing in the subthalamic area of Parkinson’s patients. Neuroimage. 2020;209:116518. 10.1016/j.neuroimage.2020.116518 31911251

[42] Luo C , Song W, Chen Q, et al Reduced functional connectivity in early-stage drug-naive Parkinson’s disease: A resting-state fMRI study. Neurobiol Aging. 2014;35(2):431–441. 24074808 10.1016/j.neurobiolaging.2013.08.018

[43] Rolinski M , Griffanti L, Szewczyk-Krolikowski K, et al Aberrant functional connectivity within the basal ganglia of patients with Parkinson’s disease. Neuroimage Clin. 2015;8:126–132. 26106536 10.1016/j.nicl.2015.04.003 PMC4473718

[44] Szewczyk-Krolikowski K , Menke RAL, Rolinski M, et al Functional connectivity in the basal ganglia network differentiates PD patients from controls. Neurology. 2014;83(3):208–214. 24920856 10.1212/WNL.0000000000000592 PMC4117363

[45] Pasos UER , Steigerwald F, Reich MM, Matthies C, Volkmann J, Reese R. Levodopa modulates functional connectivity in the upper beta band between subthalamic nucleus and muscle activity in tonic and phasic motor activity patterns in Parkinson’s disease. Front Hum Neurosci. 2019;13:223. 31312129 10.3389/fnhum.2019.00223 PMC6614179

[46] Schultz W , Romo R. Role of primate basal ganglia and frontal cortex in the internal generation of movements. *I. Preparatory activity in the anterior striatum*. Exp Brain Res. 1992;91(3):363–384. 1483512 10.1007/BF00227834

[47] Rektor I , Bareš M, Kubová D. Movement-related potentials in the basal ganglia: A SEEG readiness potential study. Clin Neurophysiol. 2001;112(11):2146–2153. 11682354 10.1016/s1388-2457(01)00662-9

[48] Litvak V , Jha A, Eusebio A, et al Resting oscillatory cortico-subthalamic connectivity in patients with Parkinson’s disease. Brain. 2011;134(2):359–374. 21147836 10.1093/brain/awq332

[49] Hirschmann J , Özkurt TE, Butz M, et al Distinct oscillatory STN-cortical loops revealed by simultaneous MEG and local field potential recordings in patients with Parkinson’s disease. NeuroImage. 2011;55(3):1159–1168. 21122819 10.1016/j.neuroimage.2010.11.063

[50] van Wijk BCM , Neumann W-J, Kroneberg D, et al Functional connectivity maps of theta/alpha and beta coherence within the subthalamic nucleus region. NeuroImage. 2022;257:119320. 10.1016/j.neuroimage.2022.119320 35580809

[51] Jenkinson N , Kühn AA, Brown P. Γ oscillations in the human basal ganglia. Exp Neurol. 2013;245:72–76. 22841500 10.1016/j.expneurol.2012.07.005

[52] Kühn AA , Trottenberg T, Kivi A, Kupsch A, Schneider GH, Brown P. The relationship between local field potential and neuronal discharge in the subthalamic nucleus of patients with Parkinson’s disease. Exp Neurol. 2005;194(1):212–220. 15899258 10.1016/j.expneurol.2005.02.010

[53] Fountas Z , Shanahan M. The role of cortical oscillations in a spiking neural network model of the basal ganglia. PLoS One. 2017;12(12):e0189109. 10.1371/journal.pone.0189109 PMC5728518 29236724

[54] Shannon CE . Communication in the presence of noise. Proceedings of the IRE. 1949;37(1):10–21.

[55] Lozano AM , Lang AE, Levy R, Hutchison W, Dostrovsky J. Neuronal recordings in Parkinson’s disease patients with dyskinesias induced by apomorphine. Ann Neurol. 2000;47(4 Suppl 1):S141–S146. 10762141

[56] Levy R , Lang AE, Dostrovsky JO, et al Lidocaine and muscimol microinjections in subthalamic nucleus reverse parkinsonian symptoms. Brain. 2001;124(10):2105–2118. 11571226 10.1093/brain/124.10.2105

[57] Belić JJ , Halje P, Richter U, Petersson P, Hellgren Kotaleski J. Untangling cortico-striatal connectivity and cross-frequency coupling in L-DOPA-induced dyskinesia. Front Syst Neurosci. 2016;10:26. 27065818 10.3389/fnsys.2016.00026 PMC4812105

[58] Ozturk M , Kaku H, Jimenez-Shahed J, et al Subthalamic single cell and oscillatory neural dynamics of a dyskinetic medicated patient with Parkinson’s disease. Front Neurosci. 2020;14:391. 32390796 10.3389/fnins.2020.00391 PMC7193777

[59] Darbin O , Dees D, Martino A, Adams E, Naritoku D. An entropy-based model for basal ganglia dysfunctions in movement disorders. Biomed Res Int. 2013;2013:742671. 10.1155/2013/742671 PMC3671275 23762856

[60] Darbin O , Adams E, Martino A, Naritoku L, Dees D, Naritoku D. Non-linear dynamics in parkinsonism. Front Neurol. 2013;4:211. 24399994 10.3389/fneur.2013.00211 PMC3872328

[61] Dresel C , Li Y, Wilzeck V, Castrop F, Zimmer C, Haslinger B. Multiple changes of functional connectivity between sensorimotor areas in focal hand dystonia. J Neurol Neurosurg Psychiatry. 2014;85(11):1245–1252. 24706945 10.1136/jnnp-2013-307127

[62] Battistella G , Simonyan K. Top-down alteration of functional connectivity within the sensorimotor network in focal dystonia. Neurology. 2019;92(16):e1843–e1851. 30918091 10.1212/WNL.0000000000007317 PMC6550502

[63] Bologna M , Berardelli A. Cerebellum: An explanation for dystonia? Cerebellum Ataxias. 2017;4:6–6. 28515949 10.1186/s40673-017-0064-8 PMC5429509

[64] Hashimoto T , Elder CM, Okun MS, Patrick SK, Vitek JL. Stimulation of the subthalamic nucleus changes the firing pattern of pallidal neurons. J Neurosci. 2003;23(5):1916–1923. 12629196 10.1523/JNEUROSCI.23-05-01916.2003 PMC6741976

