# Supplementary material for: Basal ganglia functional connectivity network analysis does not support the ‘noisy signal’ hypothesis of Parkinson’s disease
Source: Brain Commun. 2023 Apr 13;5(2):fcad123. doi: 10.1093/braincomms/fcad123 (PMC10139445; doi:10.1093/braincomms/fcad123)
Supplement: fcad123_Supplementary_Data [file fcad123_supplementary_data.zip › Supplementary_material.docx]

# Supplementary material

## Features of the LFP relationship between the GPI and the STN in the ON and OFF state with motor cues and motor response

The inter-regional network interactions were modelled as detailed in the methods section of the main text with kernel density estimates (kde) using a Gaussian. The choice of bandwidth parameters for the kernel was optimized via a linear diffusion process^1^. The discretization of the kde was over 6 by 6 grids (2^6 points). The choice of this “resolution” aimed to balance an ability to detect features of the inter-regional relationship with the number of samples present in each 100ms window at 950Hz sampling frequency and was chosen after review of higher and lower resolutions. The derived inter-regional relationships were visually inspected by examining 2 dimensional representations of the derived kde. Fluctuations in these models with time were noted on considering subtraction of a given 100ms kde model from a subsequent 100ms epoch kde model. Examination in this manner throughout the course of the trial protocol demonstrated apparent consistent changes in the modelled relationship from a baseline period prior to warning cue presentations to periods after the warning cue was presented and after the go cue triggering patient motor responses was presented, in the cued protocol, and throughout the course of movement in the self-paced protocol. This phenomenon appeared robust, observable not only within individual patient averaged responses, but on occasion in individual trial responses (supplementary material figure 1).

## Modelled interaction characteristics with self-paced movement

### GPI-STN interactions

In patients on medication deviation of the modelled network relationship between GPI and STN showed statistically significant change 1000ms prior to self-generated movements. This model deviation was maximal 50ms prior to actual movement performance (main text Figure 1 A). In contrast in untreated patients while initial model deviation occurred at the same timing maximal deviation occurred at 275ms prior to movement occurrence and the extent of model digression was less (p<0.0001, maximal 24.1% v 14.6% deviation, Wilcoxon two tailed ranks sum). Model changes appeared dominated by reductions in bivariate probability with more diffuse increases in probability in treated patients (main text Figure 1A left column, right image), but vice versa in untreated patients (right column, right image). This difference was statistically significant. (p<0.0001, mean cumulative density treated -0.03, in treated 0.01, Wilcoxon two tailed ranked sum).

**EEG-STN and EEG-GPI interactions**

Significant deviations of the modelled relationship between cortex and both the STN and GPI from baseline again preceded movement in both treated and untreated patient states as shown in the main text Figure 2A and 3A (EEG-STN treated 800ms prior, untreated 900ms prior; EEG-GPI treated 900ms prior, untreated 650ms prior). Peak deviations of the models were also observed to be around or subsequent to the time of movement other than in the untreated EEG-GPI condition (EEG-STN treated peak deviation 150ms after movement, untreated 75ms after movement; EEG-GPI treated peak deviation 50ms after movement, untreated 300ms prior to movement). It was again noted that a significant difference was observable in the extent of deviation between the two states in the EEG-STN interaction, with greater model deviation in the treated patients (p<0.0001, maximum deviation 23.6% v 7.9%, Wilcoxon two tailed ranks sum), however difference in the EEG-GPI interactions between treated and untreated states did not reach significance (main text Figure 2A, 3A). Model changes again appeared dominated by reductions in bivariate probability with more diffuse increases in probability in treated patients, but vice versa in untreated patients. This was statistically significant for both EEG-STN (p<0.0001, mean cumulative density deviation 0.004 v -0.103 untreated v treated, Wilcoxon two tailed rank sum); and EEG-GPI modelled interactions (p<0.0001, mean cumulative density deviation 0.024 v -0.001 untreated v treated, Wilcoxon two tailed rank sum).

## Modelled interaction characteristics with externally cued movement

### GPI-STN interactions

Significant model change was observed within the 75-175ms epoch after the warning cue and 125-225ms after the go cue (Supplementary Figure 2A). Go cue induced model deviation was however significantly larger than warning cue (maximal at 29.4% deviation after go cue and 21.1% after warning cue; treated, p<0.0001 for mean deviation within 1 sec of cue 21.5% v 13.3%; untreated, p<0.01 for mean deviation within 1 sec of cue 9.3% v 7.5%, Wilcoxon two tailed ranked sum test). Similar responses were seen in the untreated state however the magnitude of the maximal deviation of the model was smaller at 10.9% after warning cue, and 13.4% deviation after the go cue, with deviation in the model occurring from the 50-150ms window after both warning and go cue presentations. Again, the distinction between the magnitude of warning and go cue presentations was statistically significant, as was the distinction between model deviations in the ON and OFF states (p<0.0001 for both warning and go cue responses between states). A similar pattern regarding spatial LFP characteristics of model deviation was apparent as seen in the self-paced EEG-STN, EEG-GPI and GPI-STN relationships, in which in untreated subjects focal increases in probability exceeded reductions while the reverse was true in treated patients (Supplementary Figure 2B). When untreated (dotted line) focal increases in probability predominate after both cues, while reductions are seen in treated patients (solid line), (p=0.024, warning mean cumulative density change within 1 sec of cue 0.128 off v -0.0236 on; p<0.0001, go mean cumulative density change within 1 sec of cue 0.014 off v -0.136 on). Given that the LFP and EEG activity analyzed in the current approach retained polarity information (positive and negative deflections about 0 mean), the interpretation of the latter observations (and similar findings in the self-paced data analysis), is that the spatial characteristics of the recorded LFP were altered between treated and untreated states, and more speculatively that in the treated state there are generally higher levels of fluctuations of summative potentials at synapses and subthreshold at neuronal membranes.

# Supplementary Figure legends

**Supplementary Figure 1 Non-stationary nature of the joint probability distributions after task cues.** Images in the left column show kernel density derived estimates of the cumulative density function of STN and GPI activity in the treated state during the baseline period of the externally paced protocol. Deviations of activity from mean, in standard deviations are presented on the axes, with GPI vertically and STN horizontally. The middle column shows the difference between the baseline model and an analogous window segment after warning cue presentation. Reductions in probability are represented in blue and increases in red. The right column shows similar change after the go cue. The top row and second row show the model and cue related changes in a single trial each from subject 1 and 3. The lower row in comparison demonstrates the average response in subject 1 (n= 52). It is noted that in both individual trials and averaged responses, change is dominated by lower probability of mean activity and a more diffuse increased probability of non-mean activity.

**Supplementary Figure 2 Dynamic changes in the GPI-STN bivariate field potential model in the cued motor task.** A. Left image shows the externally cued movement associated GPI-STN model deviations from baseline in treated (solid line), and untreated (dotted line) states (n=77 treated, n=57 untreated, 2 subjects). Significant deviations from baseline are noted after both warning (W) and go (G) cue times. B. Right image demonstrates maximal positive and negative deviations of the model from baseline in epochs with significant change in treated (solid line) and untreated (dotted line) states. Similar dopamine state related model changes to those seen in self-paced movements are observed.

**Supplementary reference**

1. Botev Z, Grotowski J, Kroese D. Kernel density estimation via diffusion. *arXivorg*. 2010;38(5)doi:10.1214/10-AOS799
