# Supplementary figures and images for: Basal ganglia functional connectivity network analysis does not support the ‘noisy signal’ hypothesis of Parkinson’s disease
Source: Brain Commun. 2023 Apr 13;5(2):fcad123. doi: 10.1093/braincomms/fcad123 (PMC10139445; doi:10.1093/braincomms/fcad123)

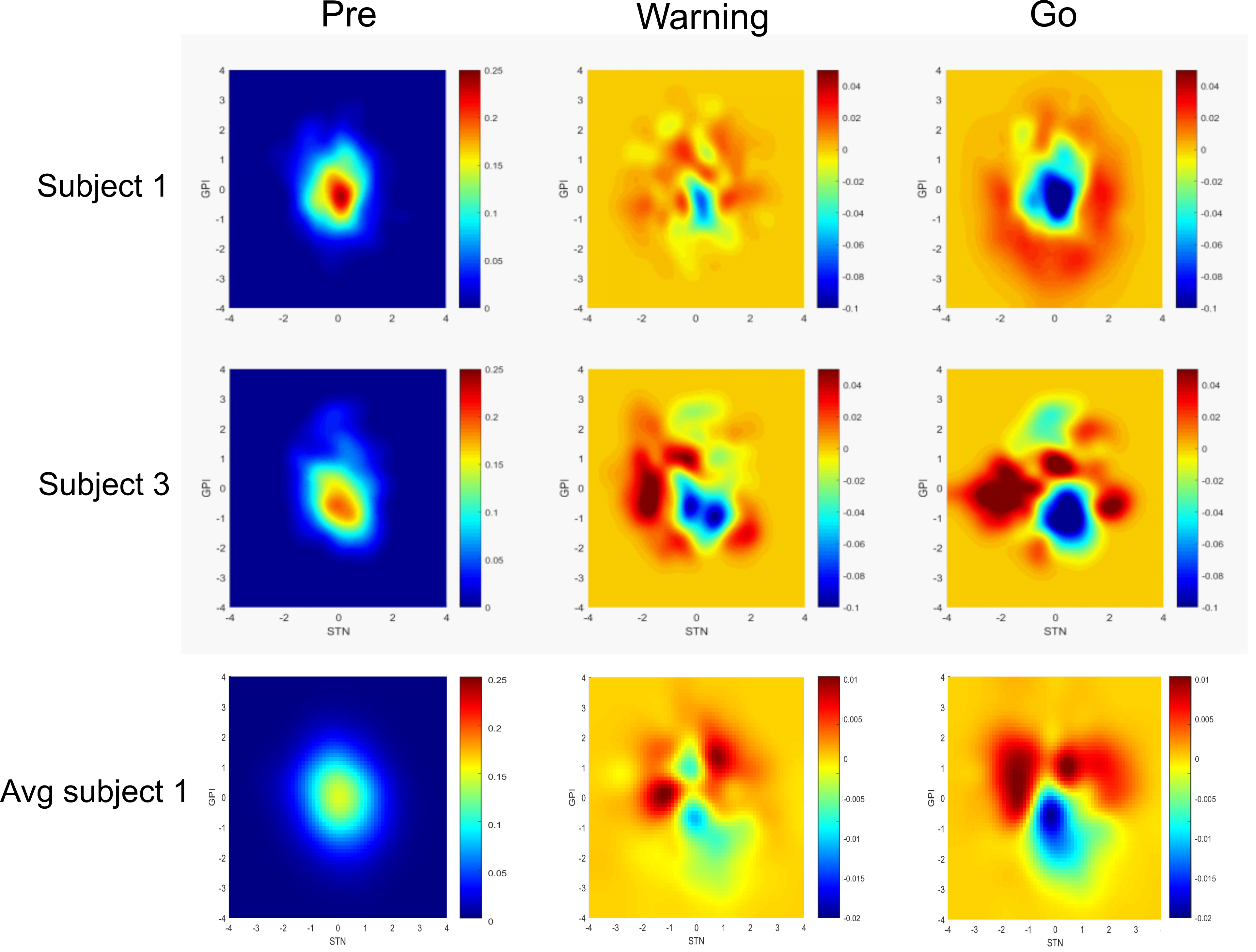

Supplement: fcad123_Supplementary_Data [file fcad123_supplementary_data.zip › Supplementary Figure 1.png]

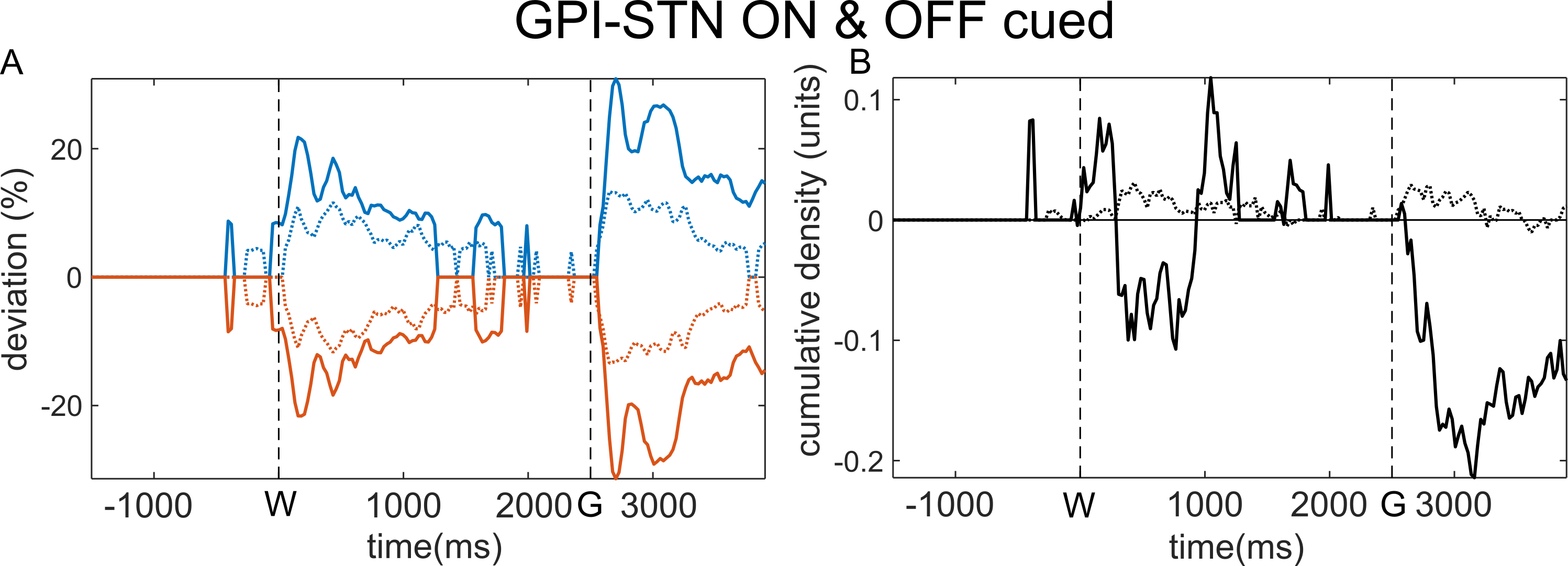

Supplement: fcad123_Supplementary_Data [file fcad123_supplementary_data.zip › Supplementary Figure 2.png]
